# GROND: automated characterization of periodic and rhythmic EEG patterns

**DOI:** 10.1088/1741-2552/aeb331

**Published:** 2026-11-03

**Authors:** Jin Jing, Chenxi Sun, Tianyu Zhang, Matthew Byrd, Alexandra–Maria Tăuţan, Lara Basovic, Peter N Hadar, Marta P Fernandes, Daniel Goldenholz, Jennifer Kim, Aaron F Struck, Sahar F Zafar, M Brandon Westover

**Affiliations:** 1 Department of Neurology, Clinical Data Animation Center, Beth Israel Deaconess Medical Center/Harvard Medical School, Boston, MA, United States of America; 2 Stanford University, Palo Alto, CA, United States of America; 3 Department of Neurology, Massachusetts General Hospital/Harvard Medical School, Boston, MA, United States of America; 4 Department of Neurology, Yale University School of Medicine, New Haven, CT, United States of America; 5 Department of Neurology, Washington University in St. Louis, St. Louis, MO, United States of America

**Keywords:** EEG, periodic discharges, rhythmic delta activity, deep learning, signal processing

## Abstract

*Objective.* Periodic discharges (PDs) and rhythmic delta activity (RDA) are common electroencephalographic patterns in critically ill patients that require detailed characterization—including lateralization, spatial localization, and frequency estimation—according to the American Clinical Neurophysiology Society 2021 standardized terminology. Manual characterization is subjective, time-consuming, and exhibits substantial inter-rater variability. This study presents a comprehensive automated system for characterizing all four major subtypes: lateralized PDs (LPDs), generalized PDs (GPD), lateralized RDA (LRDA), and generalized RDA (GRDA). *Approach.* Two complementary pipelines were developed: the PD-Profiler and the RDA-Profiler. The PD-Profiler combines a per-channel convolutional neural network (Channelpd-Net) with hemisphere-specific learned evidence traces (HemiCET-UNet), dynamic programming with an approximately-periodic prior, and discharge-locked topographic localization. The RDA-Profiler uses iterative narrowband Hilbert refinement for frequency and lateralization, with phase-locking value analysis for spatial extent. Both pipelines were trained and evaluated on 12 425 EEG segments from 11 729 unique patients. *Main results.* Four electroencephalographers—three (SZ, TZ, AS) not involved in algorithm development, plus the primary annotator (MW) — independently scored 200 stratified segments per pattern subtype, with analysis restricted to segments accepted by a majority of the four raters. The algorithm *exceeded* expert–expert (EE) inter-rater agreement on LPD frequency (mean expert–algorithm (EA) intraclass correlation coefficient (ICC) 0.931 vs. EE 0.897; paired segment-bootstrap Δ=+0.034, 95% confidence interval [+0.016,+0.055], p<0.001), and was statistically indistinguishable from EE on every other attribute tested: GPD frequency (EA ICC 0.980 vs. EE 0.973; Δ=+0.007, [−0.000,+0.017], p=0.061), GRDA frequency (Δ=−0.020, p=0.056), LRDA frequency (Δ=−0.055, p=0.435), LPD laterality (EA κ 0.945 vs. EE 0.974; Δ=−0.030, p=0.079) and LRDA laterality (EA κ 0.961 vs. EE 0.993; Δ=−0.032, [−0.087,+0.004], p=0.269). No attribute fell significantly below the EE ceiling. PD discharge timing achieved an *F*1 of 0.817 at single-sample precision (mean absolute timing error 3.1 ms at 200 Hz; median error 0 samples). Pattern lateralization area under the receiver operating characteristic curve was 0.989 for PD hemisphere (n=1276), 0.909 for LPD vs. GPD (n=7037), and 0.837 for RDA (n=4253). PD spatial localization reached 97.3% of expert inter-rater Jaccard agreement; RDA spatial extent matched EE ICC (0.371 vs. 0.373), at a ceiling set by the inter-rater noise of verbal region-counting labels. *Significance.* This work presents the first system to reach expert-level inter-rater reliability across lateralization, spatial localization, discharge timing and frequency for both periodic and rhythmic EEG patterns: it exceeded the EE ceiling on LPD frequency and was statistically indistinguishable from that ceiling on every other attribute tested, with none falling significantly below it. Agreement remains lowest, though not significantly below the ceiling, on LRDA laterality and LRDA frequency, which we identify as the priority targets for further work. In post-hoc review of discordant cases, experts judged the algorithm’s frequency estimates more accurate than the original expert labels in 94% of cases. Automated characterization can now both substitute for and improve manual annotation in critical-care EEG.

## 1. Introduction

Continuous electroencephalographic (cEEG) monitoring is widely used across acute-care settings, including neurological, medical, and surgical intensive care units (ICUs), emergency departments, and step-down units, for detecting seizures and identifying patterns associated with secondary brain injury in critically ill patients [1, 7]. Among the most clinically significant findings are periodic discharges (PDs) and rhythmic delta activity (RDA), which lie along the Ictal–interictal continuum and are associated with increased risk of seizures, neuronal injury, and poor outcomes [2, 3, 8–11]. The American Clinical Neurophysiology Society (ACNS) standardized critical-care EEG terminology, revised in 2021 [4], prescribes that PDs and RDA be described along multiple dimensions: main term (periodic vs. rhythmic), lateralization (lateralized vs. generalized vs. bilateral independent), spatial distribution (e.g. frontal predominant, hemispheric), and quantitative features including repetition frequency and regularity. Despite the standardization of terminology, applying it is a manual, expert-only task: a trained electroencephalographer must identify the pattern type, determine its spatial distribution, estimate its repetition frequency, and describe its temporal evolution from visual inspection of multi-channel EEG tracings, and inter-rater reliability (IRR) for these characterization tasks varies widely—moderate-to-good for lateralization but substantially lower for spatial extent and frequency [5, 6, 18]. The clinical similarity of LRDA to lateralized PDs further complicates pattern classification at the boundary between rhythmic and periodic phenomena [13]. The combination of subjectivity, low reliability on quantitative features, and limited overnight specialist coverage creates bottlenecks in clinical workflow and—importantly for research—makes large-scale studies of PD/RDA features impractical.

A growing body of evidence indicates that periodic and rhythmic patterns are not merely markers of underlying brain injury but can themselves contribute to neuronal damage. Microdialysis and FDG-PET studies have shown that PDs, like seizures, are accompanied by metabolic crisis and elevated glucose utilization in injured cortex [8, 9], providing direct biological evidence that these patterns impose a metabolic burden on vulnerable tissue. At the population level, our group has shown that the proportion of monitoring time a patient spends with periodic or rhythmic epileptiform activity is independently associated with worse discharge neurological outcomes. In an analysis of cEEG from 1967 critically ill patients, peak epileptiform-activity burden was independently associated with poor discharge modified Rankin Scale score; after adjustment for age, illness severity, and other clinical covariates, increasing peak burden from 0% to 100% of monitoring time raised the estimated probability of a poor outcome by approximately 35% [12]. A subsequent counterfactual matching analysis incorporating pharmacokinetic–pharmacodynamic modeling of antiseizure-medication exposure produced concordant results: untreated patients with maximum epileptiform-activity burden of $\unicode{x2A7E}75\%$ had an estimated 22 percentage-point increase in the probability of a poor outcome relative to patients with <25% burden, and even moderate but long-lasting burden (2%–10% of monitoring time) increased the probability of a poor outcome by an estimated 13.5 percentage points [11]. Together these observations are consistent with a dose-response, and plausibly causal, contribution of periodic and rhythmic epileptiform activity to neurological injury.

Time-burden alone, however, is a coarse summary of what is fundamentally a multidimensional phenomenon. Both theoretical considerations and prior experimental work suggest that, beyond *whether* a patient is in an epileptiform state, the *characteristics* of that state matter: faster discharge frequencies and broader spatial involvement plausibly impose greater metabolic demand and a greater cumulative dose of synchronous neuronal activity on vulnerable cortex [1, 8, 9, 13]. Until now, however, we have not had automated tools that can reliably quantify these features—frequency, spatial localization, lateralization, and individual discharge timing—at the data scales required for population-level studies. Building such tools is the principal motivation for the present work: only when these characteristics can be measured reproducibly and at scale will it become possible to study *how*, not merely *whether*, periodic and rhythmic epileptiform patterns predict and contribute to adverse neurological outcomes.

Prior automated systems have addressed pattern detection and classification but not the full characterization problem. Early signal-processing systems such as the ACNS-based detector of Fürbass *et al* [14] and the NeuroTrend platform [15] demonstrated feasibility of automated pattern recognition in critical care EEG; McGraw *et al* [16] introduced a method for automated quantification of PD bursts. The IIIC (Interictal–Ictal-Injury Continuum) dataset introduced by Jing *et al* [22] provides expert consensus labels for pattern classification (LPD, generalized PD (GPD), LRDA, GRDA, seizure, other) based on crowd-sourced annotations from 10 or more expert raters per segment, and deep learning models trained on this dataset—and on related datasets of epileptiform discharges [17]—achieve high classification accuracy for distinguishing among pattern types [23, 24]. None of these systems, however, addresses lateralization, spatial localization, individual discharge timing, and frequency simultaneously. The closest prior attempt is Tăuţan *et al* [25], which recently described a signal-processing approach to joint frequency and spatial-extent estimation using autocorrelation and spectral analysis; that work demonstrated the feasibility of automated characterization. In our re-evaluation on the present cohort (see section 3.2), the Tăuţan *et al* algorithm yielded Spearman frequency correlations below 0.25 for all subtypes and limited agreement with expert spatial-extent annotations.

To address these needs, we present GROND (Generalized Rhythmic and Oscillatory Neurophysiology Descriptor), an automated system for comprehensive characterization of PDs and RDA in continuous EEG. Given a 10 s EEG segment containing a periodic or rhythmic pattern, GROND automatically determines (1) lateralization (left, right, or bilateral/generalized), (2) spatial localization (which brain regions are involved), (3) frequency of the periodic or rhythmic pattern, (4) for PDs, the precise timing of each individual discharge, and (5) for PDs, whether the pattern represents bilateral independent PDs (BIPD). GROND finally produces a structured verbal description following ACNS 2021 nomenclature (e.g. ‘LPD, left-sided, at 1.5 Hz, left frontotemporal predominant’).

This work makes four contributions. First, we present a modular pipeline architecture that combines learned evidence (convolutional neural networks (CNNs)) with structured temporal inference (dynamic programming) for PD characterization, and iterative narrowband signal processing for RDA characterization. Second, we introduce discharge-locked topographic localization—a novel spatial-localization method that computes the mean voltage topography at the moment of each detected discharge, bypassing the subjective channel-counting approach used in prior work. Third, we describe a systematic ‘contest of methods’ framework in which 76 algorithm variants were compared for RDA characterization and over 30 variants for PD spatial localization, providing empirical evidence for design decisions. Fourth, we benchmark all results against independent multi-rater expert annotations with quantitative IRR analysis, enabling direct comparison of algorithm–expert (EA) agreement with expert–expert (EE) agreement across all characterization tasks.

## 2. Methods

### 2.1. Dataset and annotations

#### 2.1.1. Ethics statement

This study was conducted under protocols approved by the institutional review boards of Massachusetts General Hospital (protocols #2023P000487 and #2024P002630) and Beth Israel Deaconess Medical Center (protocols #2022P000481 and #2022P000417). Both review boards waived the requirement for informed consent for this retrospective analysis of de-identified EEG recordings. The work was carried out in accordance with the principles of the Declaration of Helsinki.

#### 2.1.2. EEG data

We drew 12 425 EEG segments from three sources (table 1), obtained by screening 13415 candidate LPD/GPD/LRDA/GRDA segments and excluding 990 during quality review. Each segment consisted of 10 seconds of 19-channel monopolar 200 Hz EEG in common average reference montage (2000 samples per channel). Classification into subtypes (LPD n=4201, GPD n=3348, LRDA n=1489, GRDA n=3387) followed the source convention: a majority of $\unicode{x2A7E}10$ IIIC expert votes for the IIIC subset, single-rater MW review for the MW-only segments, and a majority of the four expert raters for the prior Tăuţan *et al* cohort. Quality-review exclusions were applied when a segment contained muscle or motion artifact contaminating more than half the epoch, exhibited electrode dropout or mismontaging that prevented bipolar derivation, or was rejected during expert review as not representing the labeled subtype. Representative examples of each pattern type, including both clear and ambiguous cases, are shown in supplementary figure S1.

**Table 1.** Dataset statistics. The ‘Unique patients’ total (11 729) is the deduplicated count across all four subtypes; only one patient contributed segments to more than one subtype, so the total is essentially the column sum. Verified by computing the union of patient identifiers across the LPD, GPD, LRDA, and GRDA cohorts in segment_labels.csv: a single patient (anonymous identifier abn1762) appeared in two cohorts (LPD and GRDA), and no patient appeared in three or more.

	LPD	GPD	LRDA	GRDA	Total
Segments (non-excluded)	4201	3348	1489	3387	12 425
Unique patients	3955	3093	1462	3220	11 729
Expert-reviewed frequency	1530	1110	654	1386	4680
Discharge/wave timing	1372	1504	189	313	2876/502
Spatial annotations	381	264	29	182	856
IIIC crowd votes ($\unicode{x2A7E}$10 raters)	1848	1025	319	477	3669

The first data source consisted of 10 s segments from the ‘IIIC dataset’ [22]. Each of these segments had pattern classification votes from 10 or more expert electroencephalographers (3669 segments: 1848 LPD, 1025 GPD, 319 LRDA, 477 GRDA). The second source consisted of segments selected from pattern-specific clinical EEG databases and classified by a single expert rater (MW). The third source was the prior 38-patient expert clinical dataset of Tăuţan *et al* [25], annotated independently by four raters (LB, PH, SZ, MW) for pattern classification, frequency, spatial extent, and discharge timing; this cohort is used exclusively for the prior-cohort validation in section 3.2 and is distinct from the 200-segments-per-subtype canonical IRR cohort described in section 3.2.

#### 2.1.3. Annotation framework

We developed a multi-layer annotation framework with task-specific interactive review tools.

**Pattern classification and laterality.** For IIIC crowd-labeled segments, pattern subtype was assigned based on the majority vote across expert raters. For MW-labeled segments, classification was performed during visual review. Laterality was annotated as left, right, or bilateral/generalized through the interactive review viewer: for PD subtypes, by the dominant hemisphere of the discharge train; for LRDA, by the hemisphere of the rhythmic envelope. The total number of laterality annotations (left vs. right hemisphere dominance) comprised 1364 LPD segments and 1039 LRDA segments. The LRDA segments were selected from 1374 candidates, of which 338 were rejected as not meeting LRDA criteria; the rejected segments were excluded from subsequent analysis.

**Frequency.** Expert raters reviewed frequency labels for 4680 segments across all four pattern subtypes. For PD subtypes, MW annotated frequency during combined timing–frequency–laterality review. For RDA subtypes, MW reviewed frequency using an interactive viewer that displayed a default value determined by fitting a narrowband-filtered overlay; further details on the fitting method are provided below. The reviewer could confirm the default, type any other frequency judged correct, or reject the segment; MW reviewed 993 RDA segments, yielding 399 frequency corrections and 594 confirmations. We added 453 LRDA/GRDA frequency labels from segments with at least 50% IIIC crowd agreement. Three additional expert raters (LB, PH, SZ) independently annotated frequency for 1060 segments (∼265 per subtype) from the expert dataset.

**Discharge timing.** An expert (MW) annotated individual discharge times for 2876 PD segments (1372 LPD, 1504 GPD) through three rounds of iterative model-assisted review. In the first round, MW annotated discharge times de novo. These timings were utilized to develop an algorithm (the HemiCET-UNet + dynamic programming (DP), described below). In rounds 2 and 3, the algorithm generated candidate discharge times that MW reviewed and corrected, yielding 61 corrected discharge sequences and 15 rejected segments across three review cycles.

**Annotation strategy and rater coverage.** MW labeled the full corpus using the interactive tools developed for the present work; SZ, AS, and TZ each used the same interactive tools to independently label a more limited subset (described below). The two multi-rater evaluation cohorts (described below) were assembled from the labeled corpus by patient-stratified random sampling within each pattern subtype, with the canonical cohort capped at 200 segments per subtype for rater workload and the Tăuţan cohort fixed by the prior dataset. Although MW contributed to development annotations and iterative review, the principal performance claims of the study are based on independent multi-rater evaluation cohorts involving expert raters who did not participate in algorithm development.

We report two complementary IRR evaluations:

**(i) Canonical 4-rater independent-expert evaluation (MW, SZ, TZ, AS).** Three independent electroencephalographers (SZ, TZ, and AS) – none of whom contributed to algorithm development—independently scored lateralization, frequency, and discharge timing on the same 200 stratified segments per pattern subtype (LPD, GPD, LRDA, GRDA; supplementary figure S4). Each rater also recorded a per-segment accept/reject decision (rejected segments were judged by the rater not to be valid instances of the labeled subtype). The canonical IRR analysis includes only segments where the per-segment accept count exceeded the per-segment reject count (i.e. a strict majority across the four raters), removing dataset-labeling noise from the IRR computation; an individual rater’s labels are used only on segments where that rater also accepted the segment. Under this canonical inclusion rule, segment counts per task are LPD 177/200, GPD 191/200, LRDA 104/200, and GRDA 134/200; rejection rates differed by rater (MW 0%–13%, SZ 5%–44%, TZ 7%–35%, AS 15%–73%) with the highest rates on LRDA. IRR across all $\binom{4}{2} = 6$ EE pairs and all 4 expert–algorithm (EA) pairs is reported in figure 4(paired EE vs. EA bars with bootstrap 95% confidence interval (CIs) and significance tests) and in detail in the supplementary analysis document (paper_materials/independent_expert_tasks /analysis_v1.md).

**(ii) Independent prior-cohort evaluation (PH, LB, SZ, MW; Tautan *et al* [25] dataset).** As a check on a different annotation cohort, we additionally evaluated against the three expert raters (PH, LB, SZ) who labeled the Tautan *et al* dataset, with MW added as a fourth rater on the same segments. The PH/LB/SZ frequency labels were produced by visual cycle-counting on raw EEG, without the interactive narrowband-overlay tools introduced in the present work; this perceptual-task difficulty difference, not annotator skill, accounts for the higher per-rater variance on this cohort, and is reflected in lower EE intraclass correlation coefficients (ICCs) (LPD 0.50, GPD 0.47, LRDA 0.64, GRDA 0.84) relative to the canonical cohort. The algorithm’s agreement with these four raters is reported in table 7.

**Spatial extent and channel involvement.** Three expert raters (LB, PH, SZ) annotated the percentage of channels involved for 856 segments (381 LPD, 264 GPD, 29 LRDA, 182 GRDA). An additional 1980 IIIC segments received MW spatial extent annotations. Region-level spatial descriptions followed a 16-region parcellation scheme, which includes transitional zones (frontotemporal, centro-parietal, fronto-central, parieto-occipital) compatible with ACNS 2021 nomenclature.

#### 2.1.4. Evaluation framework

**Reproducibility.** The full dataset (EEG segments, expert labels, algorithm predictions, and cohort flags) is packaged as a single self-contained HDF5 bank (grond_data.h5) accompanying this manuscript and described in REPRODUCIBILITY.md in the public repository. All trained model weights, evaluation scripts, and figure-generation scripts are also included so that every figure and table in this manuscript can be regenerated from a single entry point (paper_materials/generate_all_figures.py). Reproducibility tiers per segment source, including a small number of segments whose original 10 s EEG slice could not be recovered after a 2026 repository cleanup, are documented in REPRODUCIBILITY.md along with audit logs of the recovery effort.

All evaluations used patient-stratified cross-validation to ensure no patient appeared in both training and evaluation sets. For models trained with k-fold cross-validation, 5-fold patient-stratified splits were used, and the per-segment prediction reported in all results came from the fold in which that segment’s patient was held out. For methods not requiring training (e.g. the narrowband Hilbert (NB-Hilbert) signal-processing pipeline), no patient-stratified split was needed because no parameters were learned from the data; the same NB-Hilbert hyperparameters were used at evaluation as at development, and held-out generalization was verified separately (section 3.2).

The metrics used throughout this work are: area under the receiver operating characteristic curve (AUC) for binary classification tasks (PD hemisphere, LPD vs. GPD, LRDA vs. GRDA); Spearman rank correlation (ρ) and mean absolute error (MAE, in Hz) for frequency estimation; *F*1 score at ±100 ms tolerance for discharge timing, with sensitivity, precision, and timing MAE (in milliseconds) reported as secondary metrics; the Jaccard index for PD region-set spatial localization; and the (ICC(3,1)) of Shrout and Fleiss [21] for continuous spatial extent and for IRR on continuous attributes. To enable direct comparison with prior work on the Tăuţan *et al* data, we used ICC(3,1) and percentage agreement [25] and treated the algorithm as an additional rater, enabling direct comparison of algorithm–expert agreement with EE agreement.

**Evaluation cohorts.** Two independent multi-rater evaluation cohorts are used: (i) the *canonical 4-rater independent-expert cohort* (MW, SZ, TZ, AS) of 200 stratified segments per pattern subtype, scored using the interactive tools developed for the present work and used as the primary source of EE and EA IRR comparisons (figure 4 panels (B)–(C) and section 3.2); and (ii) the *prior 4-rater Tăuţan et al cohort* (PH, LB, SZ, MW) of 38 patients, scored without the interactive narrowband-overlay tools described in this paper, used as an out-of-tooling validation cohort for the frequency analysis (table 7, figure 4 panel (A)). Neither cohort was used in any hyperparameter selection.

#### 2.1.5. Contest-of-methods development strategy

The PD-Profiler and RDA-Profiler algorithms presented herein were developed through a systematic ‘contest of methods’ in which a wide variety of candidate algorithms was implemented and compared empirically before any single approach was selected for the production pipeline. Across all subtasks (PD frequency estimation, PD spatial localization, PD discharge timing, RDA lateralization, and RDA frequency estimation), more than 300 distinct method variants were implemented and evaluated. PD frequency estimation alone progressed through nine refinement rounds comprising 192 experiments, spanning autocorrelation, fast Fourier transform (FFT), peak counting, Teager–Kaiser energy, harmonic product spectrum, matched filters, multi-montage estimators, ridge regression on log-frequency targets, and CNN-based approaches. PD spatial localization was selected from 26 contest entries spanning amplitude-based, phase-coherence-based, and CNN-probability-based methods. The RDA lateralization contest evaluated 76 algorithm variants on 4253 segments, and a separate GPD-specific frequency contest compared 25 variants. After identifying each subtask’s best-performing approach in isolation, we combined the winning components and refined the integrated pipeline. Throughout the Results section, contest entries are referenced by short codes whose letter prefixes indicate the contest cohort: **L** = single-pass laterality variants (e.g. L24); **V** = variance- or phase-locking value (PLV)-based channel-selection variants (e.g. V04, V05); **W** = two-pass iterative narrowband refinement variants (e.g. W05, the overall RDA winner, renamed NB-Hilbert in the main text); and **C** = discrete configurations of the discharge-timing pipeline. Supplementary section S1 documents the full naming convention and references the complete contest logs. This empirical, contest-driven development was essential because no single signal-processing or machine-learning paradigm proved best for all subtasks, and several methods that performed well in isolation degraded when naively combined with others. The contest is a descriptive method-selection procedure, not a formal hypothesis test: we report contest entries” point estimates so readers can see which design choices mattered, but we do not assign *p*-values across the 300+ variants. The claim about the production algorithm’s performance is supported by the prospective independent-expert evaluation (section 3.2, figure 4), in which the production system is scored against four expert raters who did not participate in algorithm development.

### 2.2. Montage

Both pipelines operate on the 18-channel longitudinal bipolar (‘banana’) derivation of the canonical 19-channel recording, following clinical convention. Because a bipolar derivation suppresses the common mode and could in principle attenuate the broad, synchronous activity that defines the generalized subtypes, we verified this choice with a controlled ablation in which the algorithm, its frozen hyperparameters, the evaluation cohort, the inclusion rule and the metrics were all held fixed and only the channel derivation varied across five conditions: bipolar, common-average monopolar, spherical-spline surface Laplacian [20], and bipolar fused with each of the latter two. The full protocol and results are given in supplementary section S3, and the outcome is summarized in section 3.7.

### 2.3. Frequency ranges and filter bands

Several distinct frequency limits appear in the descriptions that follow, and because they are easily mistaken for competing definitions of the delta band we state the convention explicitly here. The limits belong to three different categories, and only the first is a statement about physiology.

*(i) Estimator search ranges* bound the quantity being estimated. For PDs that quantity is the *repetition rate* of discrete, morphologically stereotyped discharges, not the frequency of an oscillation; ACNS 2021 assigns PDs to the 0.5–3 Hz range in routine practice, and we searched 0.3–3.5 Hz so that the range is not truncated at either end of the labeled distribution. For RDA the quantity is the frequency of a continuous rhythm, which by definition lies in the delta band (<4 Hz under ACNS 2021); we searched to 4.5 Hz because expert raters in this dataset assigned frequencies up to 4.5 Hz to segments they nonetheless scored as RDA, and truncating at 4.0 Hz would have imposed a ceiling on the estimator that the reference labels themselves did not respect. The autocorrelation estimator uses a slightly narrower lower bound of 0.33 Hz: on a 10 s window the autocorrelation must contain at least three cycles for the first peak to be identifiable, which sets a period limit of 3 s. These are the only two respects in which the PD and RDA search ranges differ, and both follow from the quantity being measured rather than from a different notion of ‘delta’.

*(ii) Filter passbands* are deliberately wider than the search range they serve, so that the transition band of the filter does not attenuate signal at the edges of the search range. The RDA pre-filter (0.3–5.0 Hz, fourth-order Butterworth, zero-phase) is therefore wider than the pass-1 band (0.5–4.5 Hz), which is in turn wider than the 0.5 Hz-half-width narrowband filter applied in pass 2. The Hilbert instantaneous-frequency samples are accepted over 0.3–4.5 Hz, and the final estimate is clipped to 0.25–4.5 Hz.

*(iii) Display filters* are used only for rendering EEG in figures (0.5–20 Hz in figures 1 and 2, 0.3–50 Hz in figures 6–9) and are chosen to match conventional clinical review settings. No estimator sees these filters.

**Figure 1.**
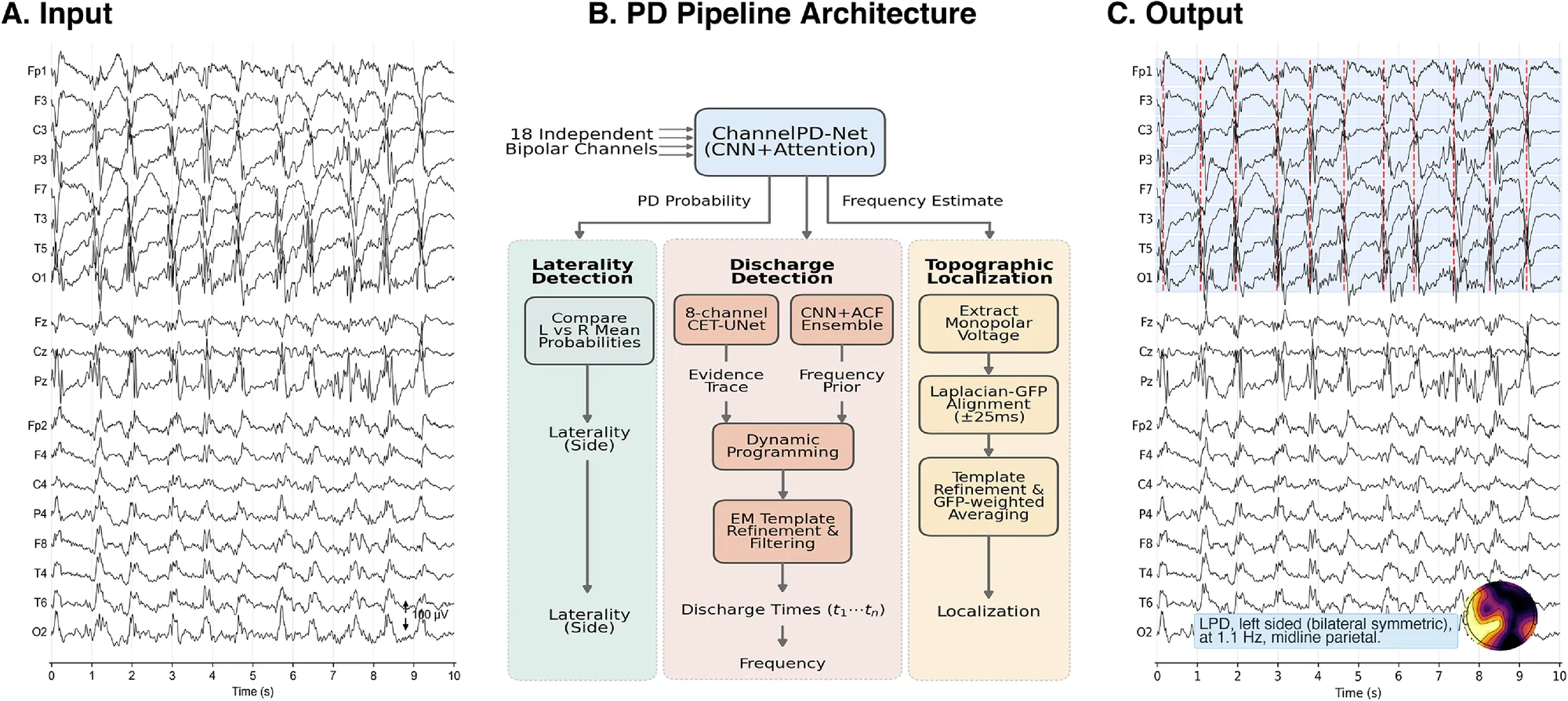
The PD-Profiler combines neural evidence with structured dynamic-programming inference to characterize PDs. Three-panel overview of the periodic discharge (PD) characterization pipeline applied to an example LPD segment. (A) Input: raw 19-channel average-reference EEG (10 s, 200 Hz). (B) Pipeline architecture: 18 independent bipolar channels are processed by ChannelPD-Net (CNN+Attention), which produces per-channel PD probabilities and frequency estimates. Three downstream modules operate in parallel: (1) laterality Detection compares left versus right hemisphere mean probabilities; (2) discharge Detection uses 8-channel HemiCET-UNet evidence and CNN+ACF frequency prior fed into dynamic programming with expectation-maximization (EM) template refinement, yielding individual discharge times and inter-peak-interval (IPI) derived frequency; (3) topographic Localization extracts monopolar voltage at discharge peaks, applies Laplacian–global field power (GFP) alignment with two-pass template refinement and GFP-squared-weighted averaging to produce a spatial localization map. (C) Output: the same EEG with red dashed lines marking detected discharge times, light blue shading on the involved hemisphere, a Laplacian topoplot showing the discharge topography, and a verbal description following ACNS 2021 standardized nomenclature. Scale bar: 100 μV.

**Figure 2.**
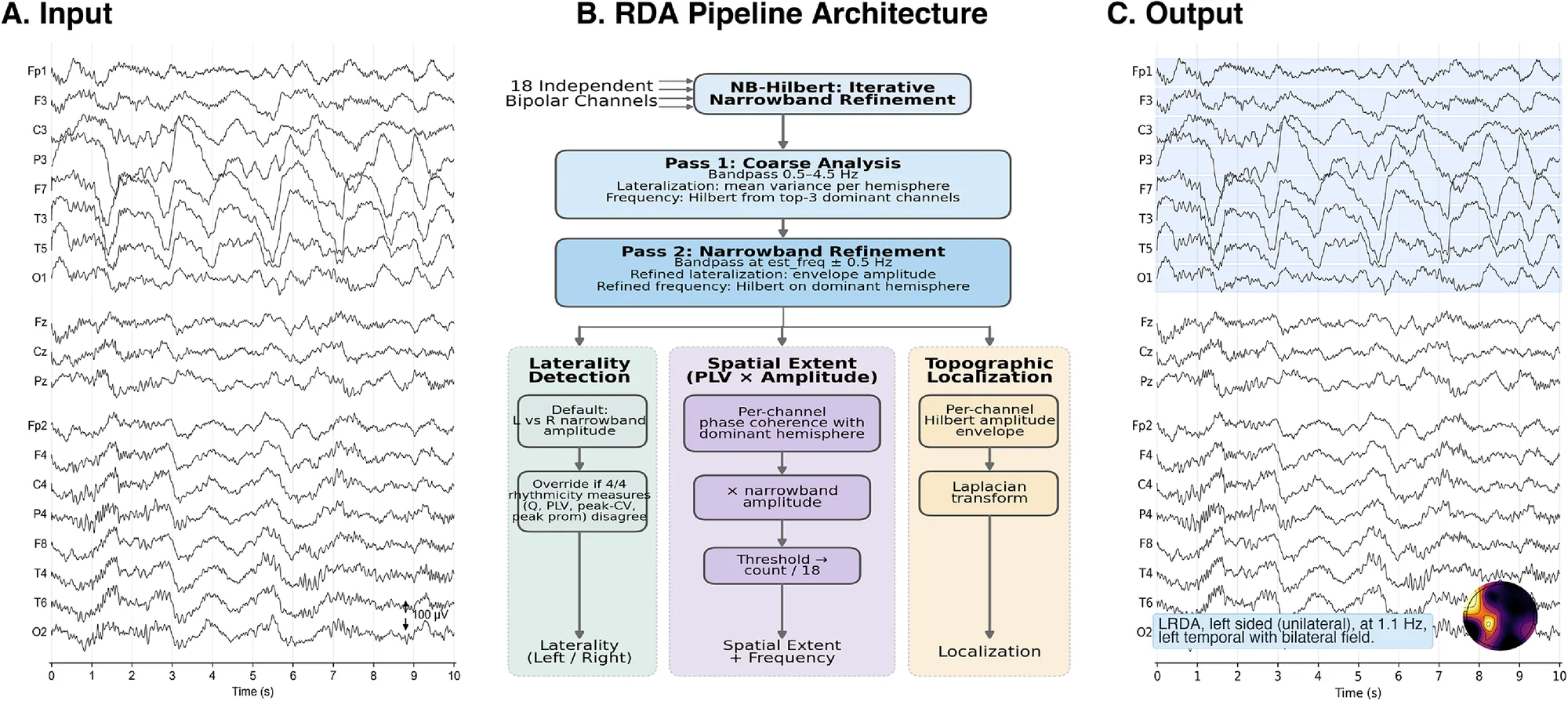
The RDA-Profiler uses two-pass iterative narrowband refinement to characterize continuous rhythmic activity. Three-panel overview of the rhythmic delta activity (RDA) characterization pipeline applied to an example LRDA segment, following the same layout as figure 1. (A) Input: raw 19-channel average-reference EEG. (B) Pipeline architecture: the NB-Hilbert iterative narrowband refinement algorithm performs two passes. Pass 1: coarse analysis with broadband (0.5–3.5 Hz) filtering, estimating lateralization via per-hemisphere mean variance and frequency via Hilbert instantaneous frequency from the top-3 dominant channels. Pass 2: narrowband filtering at the estimated frequency ±0.4 Hz, with envelope amplitude for refined lateralization and Hilbert frequency on the dominant hemisphere only. Three output branches produce laterality, spatial extent (via per-channel PLV with the dominant hemisphere), and frequency. (C) Output: the same EEG with hemisphere shading, a Laplacian amplitude topoplot, and a verbal description. No discharge markers are shown, as RDA is a continuous rhythmic pattern. Scale bar: 100 μV.

Table 2 lists every frequency limit used anywhere in the system, with its category and rationale.

**Table 2.** Every frequency limit used in the GROND system, with the category it belongs to and the reason for its value. Search ranges bound the estimated quantity; filter passbands are set wider than the search range they serve so that the filter transition band does not attenuate signal at the range edges; display filters affect rendering only and are not seen by any estimator. The apparent disagreement among these limits reflects the three different roles, not three different definitions of the delta band. Abbreviations: ACF, autocorrelation function; GFP, global field power; PD, periodic discharge; PLV, phase-locking value; RDA, rhythmic delta activity.

Limit	Category	Where used	Rationale
0.3–3.5 Hz	Search range	ChannelPD-Net frequency (section 2.4)	PD repetition rate; ACNS routine range 0.5–3 Hz, widened so the labeled distribution is not truncated
0.33–3.5 Hz	Search range	ACF frequency prior (section 2.4)	As above, but the lower bound is raised to the 3-cycle autocorrelation limit on a 10 s window (1/3 s$ ^{-1}$)
0.3–4.5 Hz	Search range	NB-Hilbert instantaneous frequency (section 2.5)	RDA oscillation frequency; delta band (<4 Hz) extended to the 4.5 Hz maximum present in the expert labels
0.25–4.5 Hz	Output clip	RDA-Profiler final estimate (section 2.5)	Range of the expert-label distribution
0.5–3.5 Hz	Filter passband	Hybrid-PLV spatial extent (section 2.4)	Contains the PD repetition rate and its immediate neighbourhood; phase coherence needs no margin beyond the pattern band
0.3–5.0 Hz	Filter passband	RDA pre-filter (section 2.5)	Wider than the 0.5–4.5 Hz pass-1 band so the transition band does not attenuate the search-range edges
0.5–4.5 Hz	Filter passband	NB-Hilbert pass 1 (section 2.5)	Matches the RDA search range
$f_1 \pm 0.5$ Hz	Filter passband	NB-Hilbert pass 2 (section 2.5)	Narrowband refinement centered on the pass-1 estimate; half-width tuned in the contest of methods
0.5–20 Hz	Filter passband	Discharge-locked topography (section 2.4)	Retains discharge morphology (sharp transients contain energy well above the repetition rate) while removing drift and muscle artefact, before the GFP alignment step
0.5–20 Hz	Display only	figures 1, 2	Conventional clinical review setting
0.3–50 Hz	Display only	figures 6–9	Conventional clinical review setting for morphology assessment

### 2.4. PD characterization pipeline (PD-Profiler)

The PD-Profiler (figure 1) processes 19-channel monopolar EEG segments in a single forward pass. From each segment it produces five outputs: lateralization, spatial localization, discharge timing, frequency estimation, and a structured verbal description. Internally, the pipeline comprises five components (table 3) operating on a shared 18-channel bipolar representation derived from the monopolar input using standard longitudinal and transverse bipolar pairs.

**Table 3.** Complete component inventory of the PD-Profiler. Every production component is listed with its architecture, its number of *learned* parameters (weights fitted by gradient descent), its number of *fixed* parameters (constants set once during the contest-of-methods development phase, section 2.1.5, and frozen thereafter), and its input and output. The six signal-processing components carry no learned parameters, and the two networks carry no fixed parameters: their only non-learned tensors are batch-normalization running statistics, which are estimated from data rather than set by hand. The dashes in the previous version of this table denoted these absences, and every count is now stated explicitly as a number. Learned-parameter counts are exact trainable-tensor counts for the shipped checkpoints. Discharge-locked topographic localization is the production spatial-output method; Hybrid-PLV was a contest entry evaluated during method selection and is described in supplementary section S2 for reference, but is *not* part of the production pipeline and is therefore not listed here. Abbreviations: CNN, convolutional neural network; TKEO, Teager–Kaiser energy operator; ACF, autocorrelation function; DP, dynamic programming; EM, expectation–maximization; GFP, global field power; HPP, handcrafted peak prior; IPI, inter-peak interval; PD, periodic discharge.

Component	Architecture	Learned param.	Fixed param.	Input → output	Role
ChannelPD-Net	1-D CNN + temporal attention	69 635	0	1×2000 samples → (p, log⁡f)	Per-channel PD detection and frequency
HemiCET-UNet	1-D encoder–decoder U-Net	129 537	0	8×2000 samples → 2000 evidence	Frame-level discharge evidence
HPP	Pointiness + TKEO	0	3	8×2000 samples → 2000 evidence	Handcrafted peak-prior evidence
Evidence combination	Product boost	0	3	2 traces → 1 trace	Fuses learned and handcrafted evidence
CNN+ACF ensemble	Weighted average	0	2	($f_\mathrm{CNN}$, $f_\mathrm{ACF}$) → $f_\mathrm{prior}$	Frequency prior for DP
Dynamic programming	Viterbi-style DP + EM refinement	0	5	evidence + $f_\mathrm{prior}$ → discharge times	Temporal inference with periodic prior
IPI frequency	Median inter-peak interval	0	0	discharge times → f	Final PD frequency estimate
Discharge-locked topo	Laplacian–GFP alignment	0	4	19×2000 + times → 19-ch map	Spatial localization (production)

#### 2.4.1. channel CNN (ChannelPD-Net)

ChannelPD-Net is a lightweight one-dimensional CNN with temporal attention pooling that operates independently on each of 18 bipolar EEG channels. Given a single 10 s *z*-scored channel (2000 samples at 200 Hz), the model jointly predicts (i) the probability that a PD pattern is present and (ii) the log-frequency of the pattern. The architecture comprises four convolutional blocks (kernel sizes 51, 25, 13, 7; channels 16, 32, 64, 64; stride 2 throughout) with batch normalization, Gaussian Error Linear Unit activation, and dropout (0.1–0.2), downsampling the temporal dimension by a factor of 16 to 125 time steps. The kernel-size schedule (large at the input to capture wave morphology at multiple temporal scales, narrowing in deeper layers) and channel widths were selected from the PD detection contest entries (section 2.1.5); we retained the configuration that produced the highest held-out validation *F*1 and did not perform a separate post-contest hyperparameter sweep. A learned temporal attention mechanism projects each 64-dimensional feature vector to a scalar logit via a 1×1 convolution, applies softmax normalization across time, and computes an attention-weighted context vector. Two independent linear heads produce a PD probability (sigmoid output) and a log-frequency estimate (linear output).

The model was trained with a multi-task loss combining binary cross-entropy for detection and mean squared error for log-frequency estimation (masked to PD-positive channels, weighted by 0.5). Training used 5-fold patient-stratified cross-validation with data augmentation including amplitude scaling (0.8–1.2×), additive Gaussian noise (20–40 dB signal-to-noise ratio, SNR), and circular time shifts (±50 samples). The 5-fold ensemble prediction was the arithmetic mean across fold models. All discharge-timing performance metrics reported in the Results section were computed from out-of-fold predictions on held-out patients under this patient-stratified evaluation framework.

**Laterality detection.** ChannelPD-Net determines hemisphere laterality by comparing the mean PD probability across left-hemisphere channels versus right-hemisphere channels in the bipolar montage, assigning the hemisphere with higher mean probability as the dominant side.

**CNN frequency estimate.** ChannelPD-Net computes the segment-level CNN frequency as a PD-probability-weighted average of per-channel log-frequency predictions, converted back to linear frequency and clipped to the physiological range of 0.3–3.5 Hz.

#### 2.4.2. Discharge timing: HemiCET-UNet and dynamic programming

**HemiCET-UNet.** HemiCET-UNet (Hemisphere-restricted Convolutional Evidence-Trace U-Net) generates discharge evidence using a one-dimensional U-Net encoder–decoder architecture with 129 537 trainable parameters. Unlike ChannelPD-Net, which provides a single summary probability per channel, HemiCET-UNet produces a dense evidence trace at the full 200 Hz resolution indicating the instantaneous likelihood of a discharge at each time point. The encoder mirrors the ChannelPD-Net backbone (four strided convolutional stages), while the decoder progressively upsamples through transposed convolutions with skip connections from corresponding encoder stages, producing a sigmoid-activated output of dimension 2000.

Training targets were constructed from expert-annotated discharge times as Gaussian peaks (σ=10 ms). The loss function combined weighted binary cross-entropy (positive weight =20 to address temporal sparsity), a sharpness penalty encouraging sparse evidence (weight 0.1), and a floor loss ensuring learned evidence at labeled discharge locations was at least as strong as handcrafted evidence (weight 0.05; see below). The model was trained with 5-fold patient-stratified cross-validation on 657 segments with ground-truth discharge timing, with data augmentation including amplitude scaling, additive noise, channel dropout (p=0.15), and discharge time jitter (σ=5 ms).

The HemiCET-UNet operates on 8-channel hemisphere input rather than the full 18-channel bipolar montage. For LPD segments, the model processes the affected hemisphere only; for GPD segments, both hemispheres are processed independently and the result with higher overall evidence is retained. This hemisphere-specific design avoids contamination from the uninvolved hemisphere in lateralized patterns and improves performance compared to full 18-channel processing.

**HPP.** Complementary evidence is computed from two handcrafted signal features. The pointiness trace captures the sharpness of transient waveforms by computing the ratio of squared prominence to half-width at half-prominence for each local maximum. The TKEO responds to instantaneous energy and frequency. Both traces are *z*-scored and combined with fixed weights (0.6 pointiness, 0.4 TKEO), Gaussian-smoothed (σ=15 ms), and clipped to non-negative values.

**Product-boosted evidence combination.** We combined the aggregated HPP and CET evidence traces using a product-boost formula that amplified regions of agreement: $E(t) = \max(E_\mathrm{hpp}, E_\mathrm{cet}) + 3.0 \cdot E_\mathrm{hpp} \cdot E_\mathrm{cet}$, where both traces are normalized to [0,1] and the CET trace is thresholded at the 80th percentile of nonzero values with a floor of 0.3. Since both inputs lie in [0,1], E(t) takes values in [0,4]; the trace is used as a relative ranking input to the DP candidate scorer and is not rescaled because the DP score depends on relative differences across candidates rather than absolute magnitude. The boost coefficient (3.0), percentile threshold (80%), and floor (0.3) were selected during the contest-of-methods development phase (section 2.1.5) by grid search over discharge-detection *F*1 on a held-out validation split.

**CNN+ACF frequency ensemble.** We obtained the frequency prior for dynamic programming by combining the CNN frequency estimate (PD-probability-weighted average across channels) with an autocorrelation-function-based estimate (ACF; first prominent peak in the normalized autocorrelation of the pointiness trace within the 0.33–3.5 Hz range), using fixed weights (0.8 CNN, 0.2 ACF), determined experimentally during the ‘contest of methods’.

**Dynamic programming.** A forward dynamic programming algorithm with an approximately-periodic prior detects individual discharge times. Candidate peaks are extracted from the combined evidence trace within an automatically detected active interval. The DP algorithm selects the optimal approximately-periodic subsequence of candidates by maximizing a score function with superlinear evidence rewards ($E(c)^{1.5}$ minus an existence cost of 0.05), quadratic penalties for deviations from the expected period (α=1.275), and skip penalties allowing up to 3 consecutive missed discharges (β=0.3 per skip); the optimal path is recovered by backtracking from the highest-scoring endpoint. Full mathematical specifications, including pseudocode and parameter values, are provided in supplementary section S2.

**Post-processing.** Discharge times are refined through three rounds of EM-style template refinement, in which the mean discharge template is cross-correlated with the full evidence trace to generate improved candidates, followed by re-running DP. A post-hoc confidence filter removes detections with evidence below 30% of the median peak evidence.

**IPI-derived frequency.** We compute the final frequency estimate as the reciprocal of the median IPI across detected discharges. This estimate is more accurate than the CNN+ACF prior because it leverages the actual detected timing sequence.

#### 2.4.3. Spatial localization

We use two complementary approaches for spatial localization of PDs.

**Hybrid-PLV spatial extent.** We score per-channel involvement using a hybrid of CNN probabilities and PLV analysis. The top-3 channels by ChannelPD-Net probability serve as references (with contralateral suppression for LPD); we bandpass-filter all channels to 0.5–3.5 Hz and compute the PLV between each channel and the circular-mean reference phase. The combined score ($0.5 \times \text{CNN probability} + 0.5 \times \mathrm{PLV}$) maps to 8 anatomical regions (left/right frontal, temporal, centro-parietal, occipital) by taking the maximum channel score per region. We classify regions exceeding a threshold of 0.4 as involved and report spatial extent as the fraction of involved regions.

**Discharge-locked topographic localization.** A novel approach to spatial localization computes the mean voltage topography at the moment of each detected discharge (figure 1(C). The 19-channel monopolar EEG is bandpass-filtered (0.5–20 Hz) and transformed to the surface Laplacian to sharpen spatial resolution. For each discharge time from the HemiCET-UNet + DP pipeline, the GFP peak is located within ±25 ms to align to the moment of maximal scalp field strength. A two-pass template refinement procedure is applied: the first pass extracts ±50 ms epochs around each GFP-aligned peak and averages them to create a template; the second pass cross-correlates each individual epoch’s GFP profile with the template to correct residual jitter. The mean topography is computed as a GFP-squared-weighted average across all refined discharge epochs, where the squared weighting strongly suppresses phantom discharges (time points where the DP algorithm interpolates a discharge at a location where no true electrographic transient exists) that exhibit low GFP near the baseline level.

The resulting 19-electrode topography vector is rendered using MNE-Python [19] spherical spline interpolation with the inferno colormap. Verbal spatial descriptions are generated by mapping electrode amplitudes to 16 anatomical, combined with ChannelPD-Net laterality to produce ACNS 2021-formatted descriptions (e.g. ‘LPD, left-sided, at 1.5 Hz, left frontotemporal predominant’).

### 2.5. RDA characterization pipeline (RDA-Profiler)

The RDA characterization pipeline, hereafter referred to as the RDA-Profiler (figure 2), characterizes LRDA and GRDA patterns using signal-processing methods operating on the same 18-channel bipolar EEG representation; its components are listed in table 4.

**Table 4.** Complete component inventory of the RDA-Profiler, in the same format as table 3. The RDA pipeline is entirely signal-processing based and contains no learned parameters; its fixed parameters are the filter edges, narrowband half-width, channel counts and thresholds specified in section 2.5, all selected during the contest of methods and frozen before any evaluation reported here. Abbreviations: NB-Hilbert, iterative narrowband Hilbert refinement; PLV, phase-locking value; RDA, rhythmic delta activity; LRDA/GRDA, lateralized/generalized RDA.

Component	Architecture	Learned param.	Fixed param.	Input → output	Role
NB-Hilbert pass 1	Butterworth + Hilbert	0	4	18×2000 → ($f_1$, side)	Coarse frequency and lateralization
NB-Hilbert pass 2	Narrowband + Hilbert envelope	0	3	18×2000 + $f_1$ → ($f_2$, side)	Refined frequency and lateralization
Asymmetry index	Amplitude asymmetry + threshold	0	1	(*L*, *R*) scores → LRDA/GRDA	Subtype classification
RDA-PLV	PLV × normalized envelope	0	2	18×2000 + $f_2$ → extent	Spatial extent
V14 laterality override	Four-measure unanimity rule	0	4	Rhythmicity measures → side	Laterality tie-break (section 3.2)

#### 2.5.1. NB-Hilbert: iterative NB-Hilbert refinement

Lateralization and frequency of RDA patterns are estimated using a two-pass iterative NB-Hilbert refinement procedure (NB-Hilbert; identified as contest entry W05 in supplementary section S1). All channels are first pre-filtered at 0.3–5.0 Hz (fourth-order Butterworth, zero-phase) to remove drift and high-frequency activity outside the search range; in the first pass they are then bandpass-filtered at 0.5–3.5 Hz (third-order Butterworth, zero-phase; 0.5–4.5 Hz in the production V12 configuration, see below). Filter bands and search ranges throughout are as tabulated in table 2. Coarse lateralization is determined by comparing mean broadband variance between left-hemisphere and right-hemisphere channel groups, and the dominant hemisphere is assigned to the side with higher variance. Coarse frequency is estimated using Hilbert-based instantaneous frequency analysis on the top-3 channels (by variance) of the dominant hemisphere, with the median of valid instantaneous frequency samples (within 0.3–4.5 Hz) taken as the estimate.

In the second pass, all channels are re-filtered using a narrow bandpass filter centered on the first-pass frequency estimate (±0.4 Hz). Lateralization is refined by comparing the mean envelope amplitude (via Hilbert transform) between hemispheres, and frequency is re-estimated from the Hilbert instantaneous frequency of the top-3 narrowband channels on the refined dominant hemisphere. LRDA versus GRDA classification is based on an amplitude asymmetry index $A = |L_\mathrm{score} - R_\mathrm{score}| / (L_\mathrm{score} + R_\mathrm{score})$, with LRDA assigned when asymmetry exceeds a threshold calibrated against expert labels. The asymmetry threshold was determined within training/development folds only, and all reported evaluation metrics were computed from held-out predictions.

NB-Hilbert was selected as the best unified (lateralization plus frequency) method from a systematic contest of 76 algorithm variants evaluated on 4253 RDA segments (1295 LRDA, 2958 GRDA; the subset of the 4731 labeled non-excluded LRDA/GRDA segments in table 1 for which valid 18-channel EEG data was available), achieving a lateralization AUC of 0.837 and frequency Spearman correlation of 0.635. Alternative methods evaluated included single-pass envelope amplitude (entry L24, AUC 0.826, no frequency output), PLV-selected channels (entry V04, AUC 0.809, frequency ρ=0.682), and automatic channel selection with frequency consensus (entry V05, AUC 0.790, frequency ρ=0.686). The iterative refinement approach provided the best lateralization performance by suppressing non-RDA activity through narrowband filtering tuned to the estimated pattern frequency. The full contest naming scheme is described in supplementary section S1.

**V12: retuned NB-Hilbert hyperparameters.** The production RDA frequency configuration, referred to as *V12* in figure captions and tables, uses NB-Hilbert with retuned hyperparameters (pass-1 bandpass 0.5–4.5 Hz, pass-2 narrowband half-width 0.5 Hz, top-3 dominant-hemisphere channels averaged, frequency cap 4.5 Hz), selected by a 192-combo grid search reported in supplementary section S1. To verify that V12’s hyperparameter selection generalizes, we re-ran the sweep on a patient-stratified random half (78 patients) of the canonical-cohort LRDA segments and re-evaluated the resulting best configuration on the held-out half (78 patients): the held-out EA mean ICC was 0.834 (MAE 0.160 Hz), essentially identical to the full-cohort in-sample ICC of 0.806. The best held-out-train configuration (p1_hi = 5.0, p2_bw = 0.6, top_k = 3, freq_cap = 4.5) gave a held-out ICC of 0.832, indicating that V12 is at or near the local optimum of the search grid for this task and that the in-sample/out-of-sample gap on this metric is negligible.

#### 2.5.2. RDA-PLV: spatial extent

Spatial extent of RDA patterns is quantified using a PLV-amplitude product metric. Each bipolar channel is narrowband-filtered at the NB-Hilbert-estimated frequency (±0.4 Hz). A reference signal is constructed as the mean of the top-3 narrowband channels on the dominant hemisphere. For each channel, the PLV with the reference is computed and multiplied by the normalized mean envelope amplitude. Channels with a PLV-amplitude product exceeding 0.15 are classified as involved, and spatial extent is reported as the fraction of involved channels. The PLV-amplitude product penalizes channels with high PLV but negligible amplitude (noise-driven phase locking) and channels with high amplitude but poor phase coherence (non-rhythmic activity).

### 2.6. BIPD detection

BIPD—defined by independent PD trains on each hemisphere with distinct timing sequences—are detected using a two-stage approach. In the first stage, the HemiCET-UNet + DP pipeline is run independently on each hemisphere, yielding two discharge time sequences. In the second stage, a gradient-boosted tree (GBT) classifier trained on features computed from the bilateral timing sequences is run. Features include the frequency ratio between hemispheres, inter-pulse interval regularity (coefficient of variation), matched fraction (proportion of temporally coincident discharges within 100 ms), phase consistency (mean resultant length of the cross-hemisphere phase relationship), and asymmetry measures (sequence length ratio, frequency asymmetry, count asymmetry).

The GBT classifier was trained on synthetic data to overcome the rarity of confirmed BIPD cases (21 out of 198 candidates screened). Synthetic BIPD examples were constructed by pairing two LPD sequences whose discharge trains were independent in timing—i.e. drawn from different patients (or from the same patient at different time points)—and assigning them to opposite hemispheres. Because the classifier learns from temporal-relationship features only (frequency asymmetry, matched fraction, phase consistency, etc), the diagnostic property of BIPD (independent left and right hemisphere discharge trains) is captured regardless of which hemisphere either source sequence was originally annotated from. Synthetic GPD examples were created by phase-shifting a single LPD sequence with random jitter (±25 ms) to simulate synchronous bilateral discharges. The full 3-way classification (LPD vs. GPD vs. BIPD) used 29 features comprising 18 per-channel PD probabilities from ChannelPD-Net plus 11 timing-derived features.

### 2.7. Statistical analysis

Because the study addressed multiple characterization subtasks with distinct output structures, evaluation metrics were selected according to the clinical interpretation objective of each task.

Lateralization and classification performance was evaluated using the AUC. For categorical inter-rater agreement on laterality calls, we additionally report Cohen’s κ.

Frequency estimation was evaluated primarily using ICC(3,1) to assess whether algorithm–expert agreement approached the EE reliability ceiling; Spearman rank correlation (ρ) and MAE against expert labels were additionally reported as complementary metrics and for comparison with prior work.

Discharge timing accuracy was evaluated primarily using *F*1 score (at ±100 ms tolerance), with sensitivity, precision, and timing MAE (in milliseconds) reported as secondary metrics.

Spatial localization was assessed using the Jaccard index (intersection over union) for PD region sets and the ICC(3,1) for spatial extent as a continuous measure.

For IRR analyses, 95% CIs were computed using bootstrap resampling (1000 iterations).

Reported evaluation n values are smaller than the corresponding label totals in table 1 for two reasons: (i) under 5-fold patient-stratified cross-validation, an out-of-fold prediction is computed only for segments whose patient appears in a held-out fold (segments from patients used only for training appear in no fold’s evaluation set), and (ii) frequency estimation is reported on the quality-filtered subset described above. The label-to-evaluation gap is therefore expected and method-specific; for example, the LPD versus GPD classifier is evaluated on the 7037 (of 7549 LPD+GPD) segments with both an out-of-fold prediction and the required random forest (RF) feature set, the PD hemisphere lateralization task is evaluated on the 1276 (of 1364 LPD-laterality-annotated) segments with both an out-of-fold ChannelPD-Net prediction and a confirmed left/right label, and frequency-quality-filtered n values are smaller than the expert-reviewed totals in table 1 because the quality filter additionally requires a single-rater MW review or three-rater agreement.

## 3. Results

### 3.1. Lateralization and subtype classification

**PD lateralization.** ChannelPD-Net achieved a hemisphere lateralization AUC of 0.989 (n=1276) for determining whether PDs were left-dominant or right-dominant, based on the difference in mean PD probability between left and right hemisphere channels (table 5). This near-ceiling performance indicated that the per-channel CNN reliably detects the lateralized nature of LPD patterns.

**Table 5.** Lateralization and classification performance. AUC is reported with the Hanley–McNeil 95% confidence interval. The 3-way and BIPD-vs.-GPD rows include only n=10 confirmed BIPD segments and are reported as preliminary; their wide CIs reflect the small BIPD sample size, not the accuracy on the other classes.

Task	Method	AUC (95% CI)	N
PD hemisphere (L vs. R)	ChannelPD-Net mean probability	0.989 [0.983, 0.995]	1276
LPD vs. GPD	RF on channel probs + features	0.909 [0.902, 0.916]	7037
3-way macro (LPD/GPD/BIPD)	RF on channel probs + timing	0.862^a^	5064
BIPD vs. GPD	Timing features (GBT)	0.937 [0.83, 1.00]^a^	2308
LRDA vs. GRDA	NB-Hilbert (iterative narrowband)	0.837 [0.823, 0.851]	4253

^a^Preliminary; only 10 confirmed BIPD segments in held-out evaluation (section 4.6).

**LPD versus GPD classification.** An RF classifier (300 trees) trained on 18 per-channel PD probabilities plus 8 handcrafted features achieved an AUC of 0.909 (n=7037) for distinguishing LPD from GPD. The features capturing spatial asymmetry in PD probability across channels are the primary drivers of classification performance.

**3-way PD classification (LPD vs. GPD vs. BIPD).** BIPD detection is a small, exploratory component of the present work: the dataset comprised 2756 LPD, 2298 GPD, and only 10 confirmed BIPD segments (21 confirmed BIPD cases were screened in total; 11 were retained for training and 10 used for held-out evaluation). The 3-way classifier incorporating timing-derived features reached a macro AUC of 0.862 (n=5064; per-class one-vs.-rest AUCs LPD 0.832, GPD 0.835, BIPD 0.920); in the binary BIPD-vs.-GPD comparison the classifier reached an AUC of 0.937 (n=2308). With only 10 held-out BIPD segments these AUC point estimates are uncertain: by the Hanley–McNeil approximation, 95% CIs are LPD [0.821,0.843], GPD [0.824,0.846], BIPD one-vs.-rest [0.804,1.000], BIPD vs. GPD [0.832,1.000], and the overall macro AUC is dominated by the wide BIPD interval. The most discriminative features for BIPD detection were the matched fraction (proportion of cross-hemisphere coincident discharges within 100 ms) and the phase consistency between left and right hemisphere discharge trains, which capture the defining characteristic of temporal independence between hemispheres. We caution that all BIPD numbers reported here are provisional; the synthetic-data training procedure (section 2.6) plausibly transfers to real BIPD because the timing-feature distinctions used by the classifier (independent vs. tightly time-locked discharge trains) are well-represented in the synthetic positive class, but a definitive evaluation will require a substantially larger held-out cohort of confirmed real BIPD segments.

**RDA lateralization (LRDA vs. GRDA).** The NB-Hilbert iterative narrowband refinement method achieved a lateralization AUC of 0.837 on 4253 segments (1295 LRDA, 2958 GRDA) in the V5 lateralization contest (table 5). This method outperformed 75 alternative approaches, including simple amplitude asymmetry (entry L24, AUC 0.826), PLV-based lateralization (entry V04, AUC 0.809), and various multi-channel Hilbert methods (AUC 0.790). The key to NB-Hilbert’s superiority is that the narrowband filtering in pass 2 removes non-RDA activity, sharpening the lateralization signal. Contest entry codes (L24, V04, etc) refer to the V5 lateralization contest naming scheme described in supplementary section S1.

### 3.2. Frequency estimation

**PD frequency.** On quality-filtered segments, the PD-Profiler (IPI-derived frequency from HemiCET-UNet + DP) achieved Spearman correlations of 0.778 for LPD (n=1155, 95% CI [0.754,0.800]; MAE =0.172 Hz, [0.159,0.185]) and 0.802 for GPD (n=1137, [0.780,0.822]; MAE =0.160 Hz, [0.101,0.223]) (table 6, figure 3). These results represent substantial improvements over the signal-processing baseline of Tautan *et al* [25], re-run on the present cohort and scored by Spearman ρ against the same expert frequency labels. The Tautan *et al* algorithm yielded ρ of 0.277 (LPD, MAE =0.505 Hz) and 0.508 (GPD, MAE =0.339 Hz) on the same labeled segments—approximately a 2.8-fold improvement on LPD and 1.6-fold on GPD. Patient-stratified paired bootstrap (1000 resamples) of $\Delta\rho = \rho_\mathrm{alg} - \rho_\mathrm{Tautan}$ rejects $\Delta\rho = 0$ at p<0.001 on both subtypes (LPD $\Delta\rho = +0.502$, 95% CI [+0.436,+0.564]; GPD $\Delta\rho = +0.294$, [+0.165,+0.495]); the corresponding MAE reductions (algorithm vs. Tautan) are +0.334 Hz and +0.179 Hz, both p<0.001.

**Figure 3.**
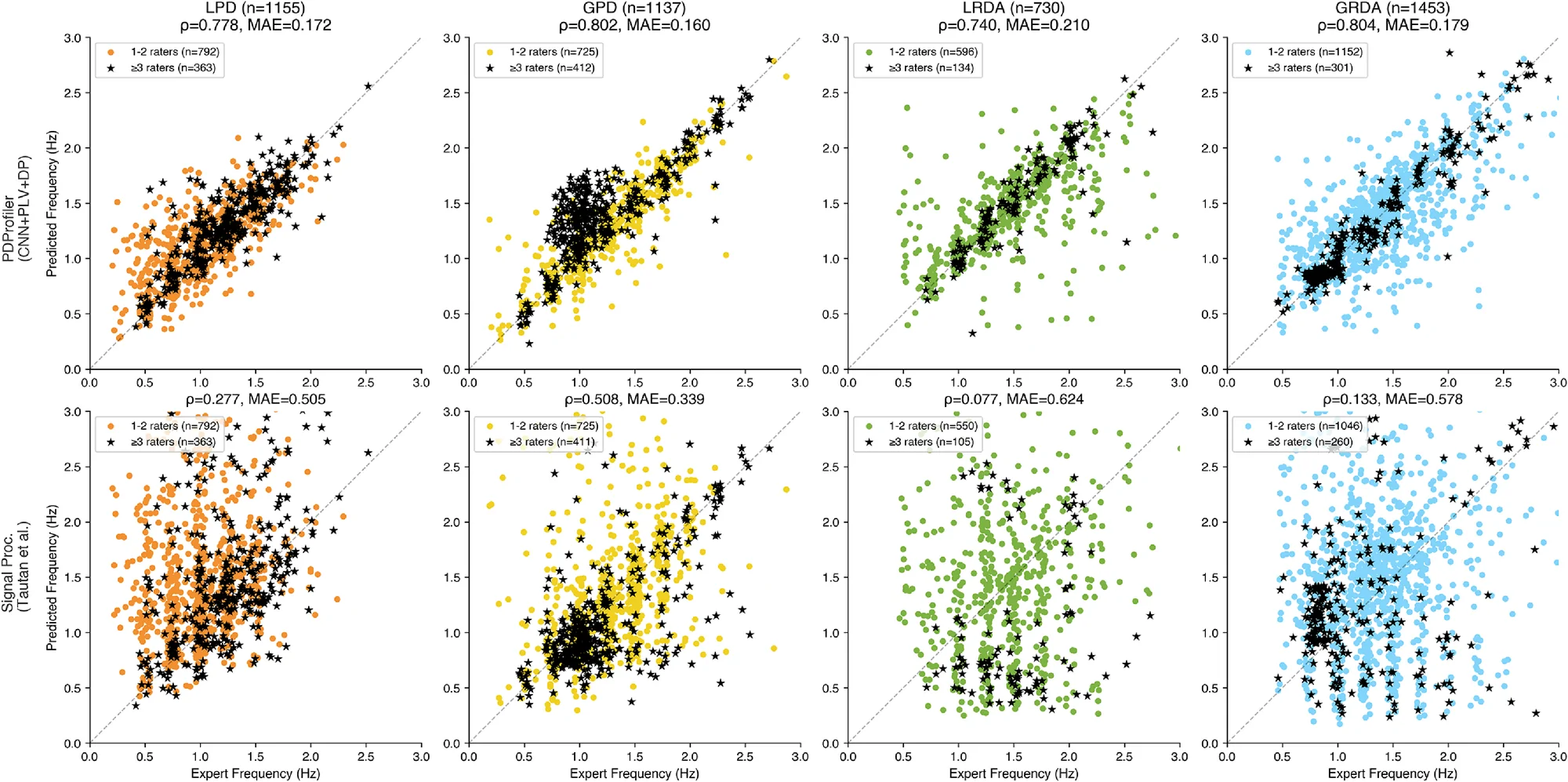
Algorithm frequency estimates correlate substantially more strongly with expert labels than the prior signal-processing state of the art. Scatter plots comparing algorithm-predicted frequency (y-axis) against expert-reviewed frequency (x-axis) for each subtype. Top row: present system (PD: CNN+ACF frequency prior refined by IPI from detected discharges; RDA: NB-Hilbert iterative narrowband Hilbert refinement, V12 hyperparameters). Bottom row: Tăuţan *et al* signal-processing baseline. Columns from left to right: LPD (n=1155; ρ=0.778), GPD (n=1137; ρ=0.802), LRDA (n=730; ρ=0.740), GRDA (n=1453; ρ=0.804). Spearman correlation (ρ) and mean absolute error (MAE, in Hz) are shown for each panel; bootstrap 95% CIs and paired Tautan-vs.-algorithm p-values are reported in table 6. Expert frequency is the mean across available rater annotations. The Tautan baseline was rerun on the same labeled cohort (per-row n in the figure title differs slightly between methods because a small minority of segments produced an estimate from one method but not the other). Dashed diagonal line indicates perfect agreement.

**Table 6.** Frequency estimation performance on the labels.csv-canonical quality-filtered cohort. Algorithm ρ 95% CIs are computed via the Fisher z-transform and algorithm MAE 95% CIs via patient-stratified bootstrap (1000 resamples). The right-most column reports the two-sided patient-stratified paired-bootstrap test of $\Delta\rho = \rho_\mathrm{alg}-\rho_\mathrm{Tautan}$ on segments where both methods produced an estimate; p<0.001 on every subtype, with bootstrap 95% CIs on $\Delta\rho$ never crossing zero (section 3.2).

Subtype	N	Algorithm ρ [95% CI]	Algorithm MAE [95% CI]	Tautan ρ	Tautan MAE	$p_{\Delta\rho}$
LPD	1155	0.778 [0.754, 0.800]	0.172 [0.159, 0.185]	0.277	0.505	<0.001
GPD	1137	0.802 [0.780, 0.822]	0.160 [0.101, 0.223]	0.508	0.339	<0.001
LRDA	730	0.740 [0.706, 0.772]	0.210 [0.184, 0.235]	0.077	0.624	<0.001
GRDA	1453	0.804 [0.785, 0.821]	0.179 [0.156, 0.198]	0.133	0.578	<0.001

The superior performance of IPI-derived frequency over direct CNN prediction ($\rho \approx 0.55$) or CNN+ACF ensemble ($\rho \approx 0.60$) confirmed that leveraging detected discharge timing for frequency estimation was substantially more accurate than spectral or regression-based approaches. The HemiCET-UNet + DP pipeline achieved a timing-derived frequency Spearman correlation of 0.837 on the 576 production evaluation cases, with a frequency MAE of 0.197 Hz.

**RDA frequency.** The NB-Hilbert iterative narrowband method (V12: pass-1 0.5–4.5 Hz, pass-2 narrowband half-width 0.5 Hz, top-3 channels, frequency cap 4.5 Hz) achieved Spearman correlations of 0.740 for LRDA (n=730, 95% CI [0.706,0.772]; MAE =0.210 Hz, [0.184,0.235]) and 0.804 for GRDA (n=1453, [0.785,0.821]; MAE =0.179 Hz, [0.156,0.198]) on quality-filtered segments (table 6, figure 3). Re-run on the same cohort and scored by Spearman ρ against expert labels, the Tautan *et al* algorithm yielded correlations of 0.077 (LRDA, MAE =0.624 Hz) and 0.133 (GRDA, MAE =0.578 Hz) – a 9.6-fold improvement on LRDA and 6.0-fold on GRDA. The corresponding paired-bootstrap differences are LRDA $\Delta\rho = +0.640$, 95% CI [+0.533,+0.738], p<0.001; GRDA $\Delta\rho = +0.655$, [+0.584,+0.718], p<0.001, with MAE reductions of +0.402 Hz and +0.390 Hz respectively (both p<0.001).

**Validation against the 4-rater Tautan *et al* cohort.** As an additional check on a different annotation cohort, we evaluated the algorithm against the four expert raters (PH, LB, SZ, MW) on the Tautan *et al* [25] dataset. PH/LB/SZ produced their labels by visual cycle-counting on raw EEG, without access to the interactive narrowband-overlay tools introduced in the present work; the resulting EE ceiling on this cohort is correspondingly lower as a consequence of the perceptual-task difficulty difference, not of annotator skill. Mean agreement across the six within-role EE pairs and the four EA pairs (table 7) shows that the algorithm meets or exceeds EE reliability on three of the four subtypes: mean EA ICC 0.74 vs. mean EE ICC 0.64 on LRDA, mean EA 0.88 vs. mean EE 0.84 on GRDA, and mean EA 0.57 vs. mean EE 0.50 on LPD. On GPD the algorithm is approximately at the EE level (mean EA 0.43 vs. mean EE 0.47); per-pair detail shows that the algorithm tracks SZ and MW tightly (ICC 0.83 and 0.85 respectively) but disagrees more substantially with PH and LB (ICCs 0.06 and −0.07), suggesting a scoring-approach difference on this subtype that warrants future investigation. The Tautan *et al* signal-processing baseline is uniformly weaker than the present algorithm on this cohort (mean expert–Tautan ICC: LPD 0.10, GPD 0.36, LRDA −0.21, GRDA 0.17).

**Validation against 4 new independent raters.** The independent-expert task described above included 200 segments per subtype (LPD, GPD, LRDA, GRDA), each scored independently by MW, SZ, TZ, and AS. Under the canonical majority-accept consensus rule (per-segment accepts > per-segment rejects across the four raters), per-task eligible segment counts are LPD 177, GPD 191, LRDA 104, GRDA 134; per-pair sample sizes within each task are reported in figure 4.

For LPD frequency, the four EA ICCs are 0.875 (MW), 0.976 (SZ), 0.923 (TZ), and 0.950 (AS) for a mean of 0.931, against a mean EE ICC of 0.897 across the six EE pairs; the difference is significantly favorable to the algorithm (Δ=+0.034, 95% CI [+0.017,+0.054], p<0.001). For GPD frequency, EA ICCs are 0.963 (MW), 0.987 (SZ), 0.977 (TZ), and 0.994 (AS) for a mean of 0.980, slightly above the mean EE ICC of 0.973, but with a CI that touches zero and a p-value at the significance boundary (Δ=+0.007, 95% CI [−0.000,+0.017], p=0.061); we therefore read GPD frequency as indistinguishable from the expert ceiling rather than above it. For GRDA frequency, EA ICCs are 0.903 (MW), 0.989 (SZ), 0.899 (TZ), and 0.913 (AS) for a mean of 0.926 against EE 0.946 (Δ=−0.020, 95% CI [−0.047,+0.000], p=0.056, n.s.).

For LRDA, the algorithm reached LRDA EA ICCs of 0.829 (MW), 0.882 (SZ), 0.852 (TZ), and 0.831 (AS) for a mean of 0.848 against EE mean 0.903 (Δ=−0.055, 95% CI [−0.195,+0.036], p=0.435, n.s.). For LRDA laterality, the original W05 rule lateralizes by amplitude (which hemisphere has more narrowband envelope amplitude at the estimated rhythm frequency); this is fallible by construction whenever artifact, slow drift, or asymmetric volume conduction inflates one hemisphere’s amplitude even when the rhythm itself is on the other side. We therefore developed V14, a hybrid that defaults to the amplitude rule but overrides it whenever four amplitude-normalized rhythmicity measures (per-channel *Q*-factor, within-hemisphere PLV, narrowband peak-amplitude consistency, and spectral peak prominence) *unanimously* disagree with the amplitude call. The resulting LRDA-laterality EA κ’s are 0.942 (MW), 0.979 (SZ), 0.960 (TZ), and 0.962 (AS) for a mean of 0.961 against EE mean 0.993 (Δ=−0.032, 95% CI [−0.087,+0.004], p=0.269, n.s.). The V14 override closes most of the gap left by the amplitude-only rule, which reached a mean κ of only 0.912 on the same segments (Δ=−0.081, p=0.035); under the production V14 rule the algorithm is numerically below the EE ceiling on this task but no longer significantly so.

To formally test whether the algorithm–expert agreement differs from EE agreement on each task, we performed a paired segment-level bootstrap (2000 resamples) of $\Delta = \mathrm{mean}_\mathrm{EA} - \mathrm{mean}_\mathrm{EE}$ (sign-corrected so positive favors the algorithm), computed on the production configuration (V12 frequency, V14 laterality). The algorithm exceeded the EE ceiling on LPD frequency (ICC Δ=+0.034, p<0.001; Spearman Δ=+0.041, p<0.001), and was statistically indistinguishable from the expert ceiling on every other agreement metric tested: GPD frequency (Δ=+0.007, p=0.061), GRDA frequency (p=0.056), LRDA frequency (p=0.435), LPD laterality (p=0.079) and LRDA laterality (p=0.269). No task fell significantly below the expert ceiling on ICC, Spearman or κ. The algorithm was significantly behind on RDA *MAE* (LRDA p=0.001, GRDA p<0.001). Because three of these p-values sit near 0.05, we repeated the GPD-frequency, GRDA-frequency and LRDA-laterality tests at 20 000 resamples under three independent seeds and confirmed that the verdicts are stable (GPD p=0.052–0.055; GRDA p=0.052–0.057; LRDA laterality p=0.26–0.27). The MAE gap reflects a small constant offset between algorithm and expert frequency estimates that is invisible to ICC and Spearman; the algorithm tracks the rank ordering of expert frequencies tightly but with a ∼0.05 Hz residual bias on RDA tasks. The LRDA-laterality gap is small in absolute terms (mean EA κ=0.961 against a 0.993 EE ceiling) and is not statistically significant. A summary visualization with paired EE/EA bars, 95% CIs on the means, and significance markers is shown in figure 4.

**Figure 4.**
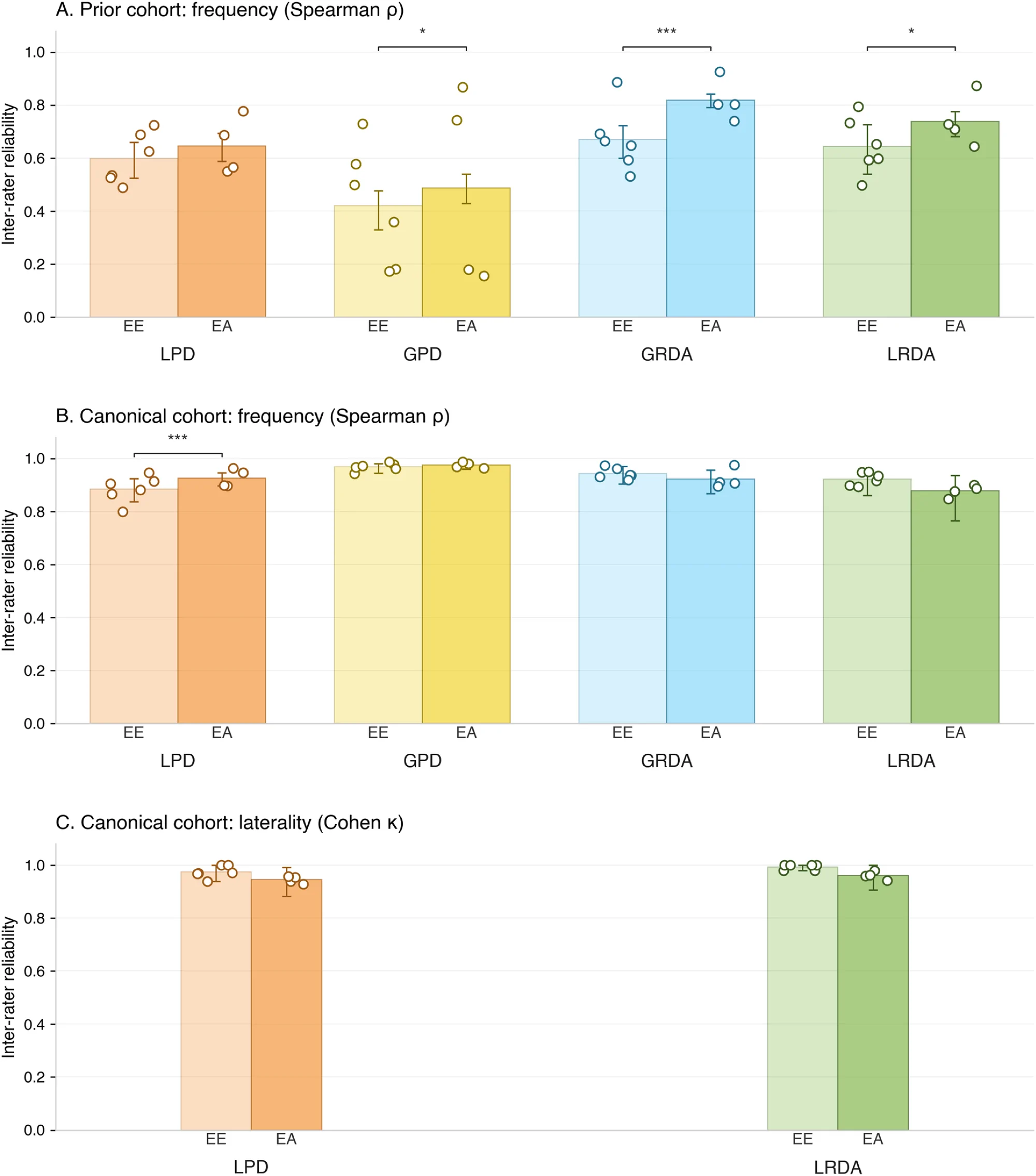
Algorithm–expert agreement matches or exceeds the expert–expert ceiling on two independent 4-rater cohorts. Within each task the left bar shows mean expert–expert (EE) agreement across all $\binom{4}{2} = 6$ within-cohort expert pairs, and the right bar shows mean expert–algorithm (EA) agreement across all 4 expert/algorithm pairs. Bar height is the mean across pairs computed by paired segment-level bootstrap (1000 resamples); error bars are 95% bootstrap CIs on that mean. Open circles overlaid on each bar are the per-pair point estimates (6 EE points, 4 EA points), with horizontal jitter for legibility. Bar color encodes IIIC pattern subtype using the same palette as figure 3(LPD orange, GPD yellow, LRDA olive-green, GRDA sky-blue); the lighter shade is EE and the darker shade is EA. Significance brackets (∗ p<0.05, ∗∗ p<0.01, ∗∗∗ p<0.001) are drawn only over comparisons where mean(EA)−mean(EE) reached significance under the paired bootstrap; non-significant comparisons are not annotated and are reported in the text; under the production configuration no comparison in panel C reached significance, and in panel B only LPD frequency did. Significance values are taken from the same paired bootstrap (2000 resamples) that produced the statistics in section 3.2, computed on the identical algorithm configuration that generated the bars. In every annotated comparison the algorithm direction was positive (EA > EE). (A) Prior 4-rater Tautan *et al* [25] cohort (PH, LB, SZ, MW): frequency Spearman ρ. PH, LB, and SZ produced their labels without the present interactive narrowband-overlay tools, so the EE ceiling on this cohort is correspondingly modest; laterality is not shown because PH and LB did not annotate laterality. (B) Canonical 4-rater independent-expert cohort (MW, SZ, TZ, AS) on 200 stratified segments per pattern subtype, restricted to segments where per-segment accepts exceeded rejects: frequency Spearman ρ. (C) Canonical cohort laterality (Cohen κ) for LPD and LRDA only (GPD and GRDA are bilateral by definition). The algorithm uses V12 frequency estimation (NB-Hilbert with retuned hyperparameters: pass-1 0.5–4.5 Hz, pass-2 narrowband half-width 0.5 Hz, top-3 dominant-hemisphere channels averaged, frequency cap 4.5 Hz) and the V14 amplitude/rhythmicity hybrid laterality rule (default to the V12 amplitude call; override only when four amplitude-normalized rhythmicity measures—per-channel *Q*-factor, within-hemisphere PLV, narrowband peak-amplitude consistency, and spectral peak prominence – *unanimously* disagree with the amplitude call). Per-pair ICCs and MAEs are reported in the main text and in table 7.

**Table 7.** Algorithm validation on the prior 4-rater cohort (raters PH, LB, SZ, MW) labeled on the Tautan *et al* [25] dataset. Mean across the within-role pairs ($\binom{4}{2} = 6$ EE pairs, 4 EA pairs, 4 expert–Tautan pairs). The PH/LB/SZ labels were produced by visual cycle-counting on raw EEG without the interactive narrowband-overlay tools introduced here, and the resulting EE ceiling on this cohort reflects that perceptual-task difference rather than annotator skill.

	Expert–Expert			Expert–Algorithm			Expert–Tautan		
Task	ICC	ρ	MAE	ICC	ρ	MAE	ICC	ρ	MAE
LPD freq	0.50	0.62	0.29	0.57	0.63	0.25	0.10	0.23	0.50
GPD freq	0.47	0.41	0.26	0.43	0.48	0.31	0.36	0.38	0.31
LRDA freq	0.64	0.71	0.22	0.74	0.73	0.20	−0.21	0.15	0.74
GRDA freq	0.84	0.76	0.16	0.88	0.84	0.17	0.17	−0.03	0.57

### 3.3. Discharge timing

On 576 evaluation cases at ±100 ms tolerance, the HemiCET-UNet + DP pipeline achieved a discharge detection *F*1 of 0.817 (sensitivity 0.884, precision 0.759; table 8). The 576 evaluation cases were a quality-filtered subset of the 657 training segments described in section 2.4, excluding cases without a documented discharge-timing review pass. The mean absolute timing error was 3.1 ms (median error 0 samples), indicating that detected discharges were localized to within a single sample (5 ms at 200 Hz) of the expert-annotated timing. The IPI-derived frequency from detected discharges achieved Spearman ρ of 0.837 with expert-reviewed frequency.

**Table 8.** Discharge-timing performance of the production PD-Profiler and three comparison configurations, on 576 evaluation segments with expert-annotated discharge times. A detection counts as a true positive when it falls within ±100 ms of an expert-marked discharge; *F*1 is the harmonic mean of sensitivity and precision under that matching. Frequency ρ is the Spearman rank correlation between the inter-peak-interval-derived frequency and the expert-reviewed frequency on the same segments. Timing MAE is the mean absolute difference between each matched detection and its expert-marked time. All values are out-of-fold predictions under 5-fold patient-stratified cross-validation, so no segment was scored by a model trained on the same patient. Rows: the production pipeline; an oracle given the expert frequency and handcrafted evidence only; the same pipeline run on all 18 bipolar channels instead of the 8 hemisphere-restricted channels; and a per-hemisphere baseline without dynamic-programming inference. Abbreviations: DP, dynamic programming; *F*1, *F*1 score; HPP, handcrafted peak prior; MAE, mean absolute error.

Method	*F*1	Sensitivity	Precision	Freq ρ	Timing MAE (ms)
HemiCET-UNet + DP (production)	0.817	0.884	0.759	0.837	3.1
Oracle (expert freq + HPP)	0.578	0.569	0.799	0.910	10.9
Full 18-channel pipeline	0.684	0.722	0.730	0.768	17.7
Per-hemisphere baseline	0.586	0.724	0.715	0.778	19.4

### 3.4. Spatial localization

#### 3.4.1. PD spatial localization

**Discharge-locked topographic localization.** The primary spatial localization method for PDs computed the mean discharge topography using the two-pass Laplacian–GFP alignment procedure described in section 2.4. Representative topographic maps are shown in figures 6 and 7. The Laplacian transform sharpened spatial resolution by removing volume-conducted contributions, producing focal peaks corresponding to cortical generators. The GFP-squared weighting reduced the effective contribution of phantom discharges by a factor of 4–10 relative to uniform averaging, and the two-pass alignment reduced cross-epoch jitter by approximately 8–12 ms compared to single-pass GFP alignment.

**Spatial extent (Jaccard agreement).** The Hybrid-PLV spatial method was evaluated against three expert raters (LB, PH, SZ) on PD segments with 3-rater ground truth (table 9; per-subtype scatter in supplementary figure S2). The choice of binarization threshold influenced model–expert agreement (supplementary figure S3); at the optimized threshold of 0.38, the mean model–expert Jaccard index was 0.731 (±0.076), compared to a mean EE Jaccard of 0.751 (±0.025), representing 97.3% of human inter-rater agreement. Notably, the model-PH agreement (Jaccard 0.837) exceeded all EE pairs (maximum: LB–SZ at 0.773), indicating that the model acted as a ‘virtual fourth rater’ with agreement patterns within the range of expert variation.

**Table 9.** Pairwise agreement on periodic-discharge spatial extent, expressed as the Jaccard index (intersection over union) of the sets of involved anatomical regions. Rows and columns are the three expert raters (LB, PH, SZ) and the Hybrid-PLV algorithm, which is treated here as a fourth rater; the matrix is symmetric and the diagonal is 1 by construction. Region involvement was binarized at the score threshold 0.38, selected by the sweep in supplementary figure S3. Evaluated on the PD segments carrying 3-rater spatial ground truth. Summary: the mean algorithm–expert Jaccard was 0.731 (±0.076) against a mean EE Jaccard of 0.751 (±0.025), i.e. 97.3% of the human inter-rater ceiling; the algorithm–PH pair (0.837) exceeded every EE pair (highest LB–SZ, 0.773). Abbreviations: PD, periodic discharge; PLV, phase-locking value.

	LB	PH	SZ	Model
LB	1.000	0.762	0.773	0.693
PH	0.762	1.000	0.716	0.837
SZ	0.773	0.716	1.000	0.662
Model	0.693	0.837	0.662	1.000

At threshold =0.62 for the per-channel involvement decision, the PD-Profiler spatial ICC was 0.764 (figure 5), below the three-rater EE ICC of 0.845 on the same segments. Per-subtype, PD-Profiler ICC matched the EE consensus on LPD (0.77 vs. 0.77). On GPD, the per-subtype EE ICC was indistinguishable from zero (0.00), indicating that the GPD spatial-extent labeling task as currently annotated is not a measurable quantity in the technical sense—expert raters did not agree on it beyond chance—so the algorithm’s GPD ICC of 0.09 should not be read as ‘the algorithm did not improve on experts’ but as a finding that the underlying labeling task lacks the inter-rater signal needed to support any ICC-style comparison on this subtype. We retain the result for completeness and discuss the implications for spatial-extent reporting in section 4.4. Tautan *et al* [25] achieved the same combined PD spatial ICC of 0.764. The Jaccard analysis above (table 9, threshold 0.38) is the more interpretable PD-spatial summary on this dataset; ICC at the channel-fraction threshold above is dominated by the GPD subset where verbal region-counting agreement is poor in the first place.

**Figure 5.**
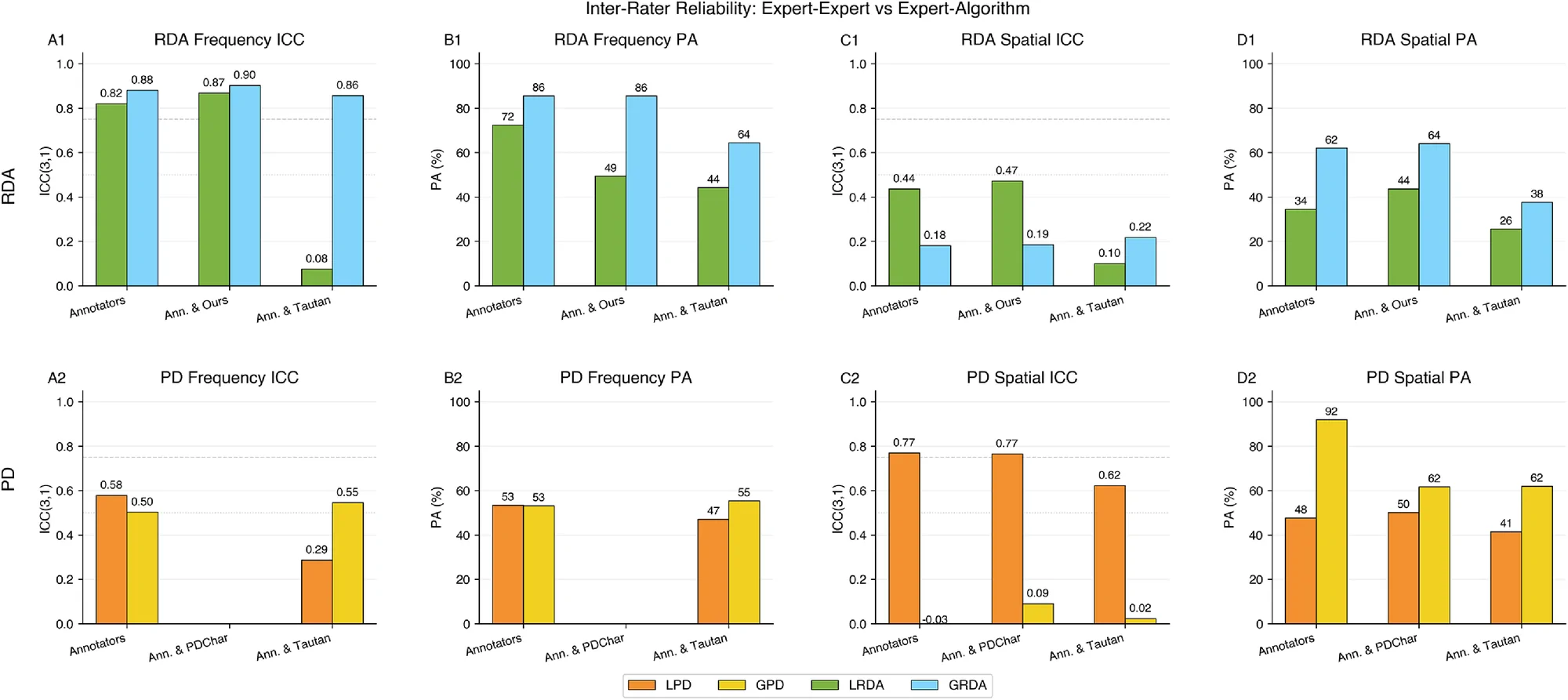
Inter-rater reliability on the prior three-rater cohort: expert–expert agreement compared with algorithm–expert agreement and with the prior signal-processing state of the art. Bars show the intraclass correlation coefficient ICC(3,1) (two-way mixed effects, single rater, consistency; Shrout and Fleiss [21]) and percentage agreement, each averaged over all rater pairs of the corresponding type, for frequency estimation and for spatial extent. Top row: RDA (LRDA and GRDA segments combined). Bottom row: PD (LPD and GPD segments combined). Three conditions are compared in every panel: expert–expert agreement among the three human raters (blue), the present system treated as an additional rater (orange), and the Tăuţan *et al* [25] signal-processing baseline treated the same way (green); scoring the algorithm as an extra rater is what makes the algorithm–expert and EE bars directly comparable. Key results: for RDA frequency the RDA-Profiler (NB-Hilbert) reached ICC =0.860 against an EE ceiling of 0.852; for PD spatial extent the PD-Profiler reached ICC =0.852 at the optimized binarization threshold against an EE ceiling of 0.845. In both cases the algorithm matched or slightly exceeded the human ceiling, and in both cases the Tăuţan *et al* baseline fell below it. The formally powered significance testing of algorithm versus expert agreement is reported on the canonical four-rater cohort in figure 4 and section 3.2; this figure is descriptive and no significance tests are performed on it. Abbreviations: EE, EE; GPD, generalized PDs; GRDA, generalized rhythmic delta activity; ICC, intraclass correlation coefficient; LPD, lateralized periodic discharges; LRDA, lateralized rhythmic delta activity; NB-Hilbert, iterative narrowband Hilbert refinement; PD, periodic discharges; RDA, rhythmic delta activity.

#### 3.4.2. RDA spatial extent

The RDA-PLV method achieved an ICC of 0.371 when the algorithm was treated as a fourth rater alongside the three experts, compared to an EE ICC of 0.373 (n=211 segments with 3-rater spatial ground truth). The algorithm MAE of 0.215 was better than some EE pairs (e.g. LB vs. SZ MAE =0.484). Tautan *et al* achieved an RDA spatial ICC of 0.215, substantially below both expert agreement and the present method.

The low absolute ICC values (approximately 0.37) for RDA spatial extent—for both the algorithm and expert raters—reflected the inherent difficulty of the verbal region-counting task. Unlike PD spatial localization, where discrete discharges create identifiable transients, RDA produces continuous rhythmic activity whose boundaries are gradual and subjective. The near-parity between algorithm and expert ICC indicated that the PLV-amplitude method captured the same information experts used when judging spatial extent. We treat the low absolute reliability as evidence that the categorical labeling task is itself noisy rather than as a target to be chased further; the principled spatial output of the system is the discharge-locked topographic map, as discussed in section 4.4.

### 3.5. End-to-end characterization examples

Figures 6–9 present representative characterization examples for all four subtypes at three difficulty levels (easy, medium, hard), selected to span the range of inter-rater agreement. Each example shows the 19-channel EEG with pipeline outputs overlaid: detected discharge times (red dashed lines, PD only), hemisphere shading (light blue), Laplacian topographic map (inferno colormap), and ACNS 2021 verbal description.

**Figure 6.**
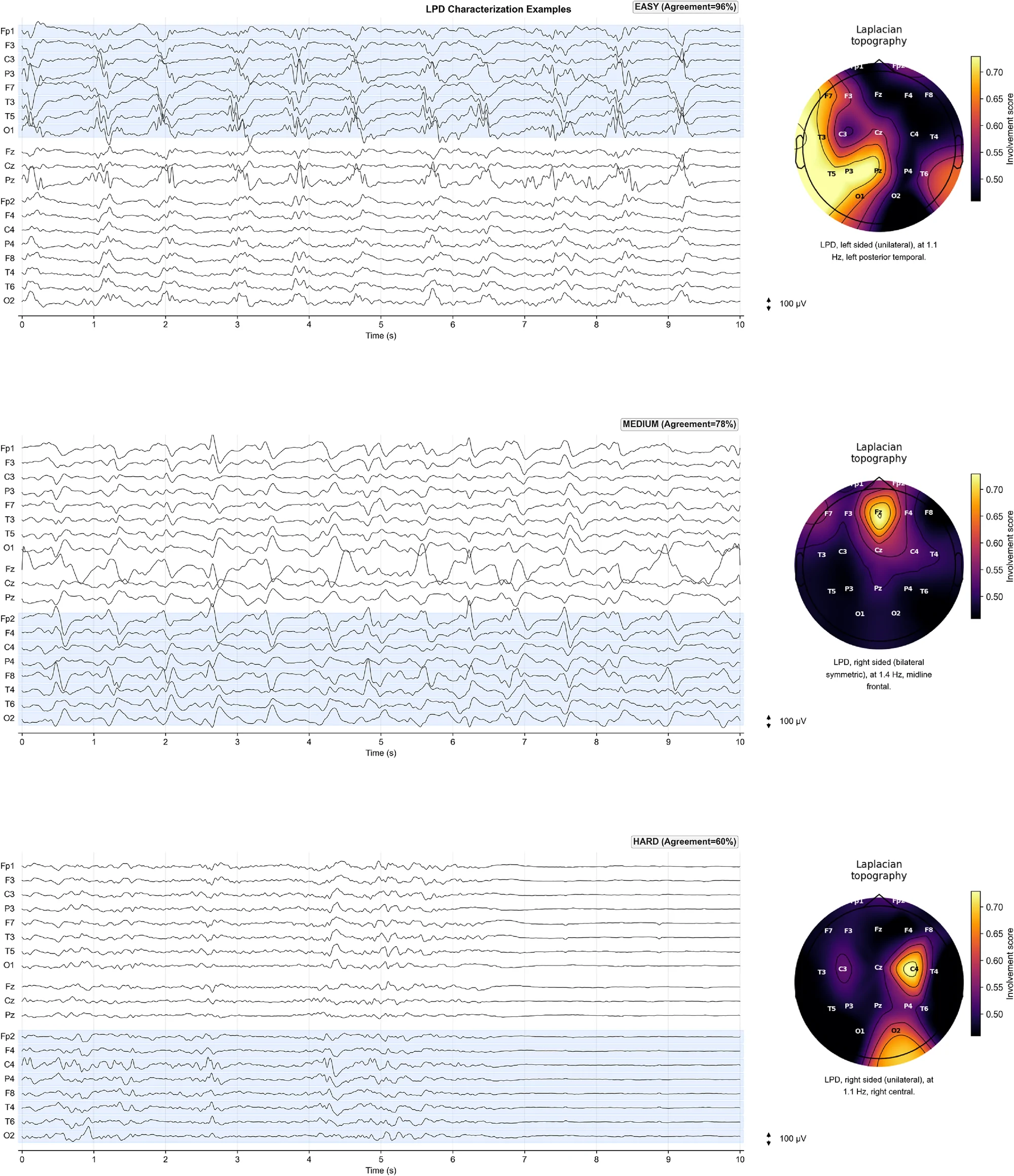
LPD characterization examples at three difficulty levels. Three representative lateralized periodic discharge (LPD) cases selected to span the range of inter-rater agreement. Each row shows one case with: (left) 19-channel average-reference EEG (10 s, bandpass 0.3–50 Hz, amplitude clipped at ±250 μV) with detected discharge times marked as red dashed vertical lines on the involved hemisphere and light blue shading indicating the lateralized side; (right) Laplacian topoplot (inferno colormap) with electrode labels, and ACNS 2021 verbal description. Top: easy case (agreement $\unicode{x2A7E}95\%$). Middle: medium case (agreement 70%–80%). Bottom: hard case (agreement 45%–60%). Agreement is the percentage of IIIC expert raters assigning the labeled subtype, and difficulty tiers were defined on that percentage before any algorithm output was inspected. Scale bar: 100 μV. Abbreviations: ACNS, American Clinical Neurophysiology Society; GFP, global field power; IIIC, ictal–interictal–injury continuum; LPD, lateralized periodic discharges.

For LPD examples (figure 6), the easy case (IIIC agreement $\unicode{x2A7E}95\%$) exhibits clear, stereotyped discharges with unambiguous lateralization and a sharp focal maximum in the topographic map. The medium case (70%–80% agreement) shows recognizable periodicity with some morphological variability; the pipeline correctly lateralizes and detects most discharges, though one or two marginal peaks are missed. The hard case (45%–60% agreement) features subtle, atypical discharges where expert raters themselves disagree; the pipeline produces a reasonable characterization, though the topographic map was more diffuse, reflecting the ambiguity of the pattern.

For GPD examples (figure 7), discharge markers span both hemispheres, and the topographic maps shows bilateral, often frontally predominant distributions. For LRDA and GRDA examples (figures 8 and 9), no discharge markers are shown (RDA being a continuous rhythmic pattern), but hemisphere shading and amplitude topographic maps correctly identify the spatial distribution.

**Figure 7.**
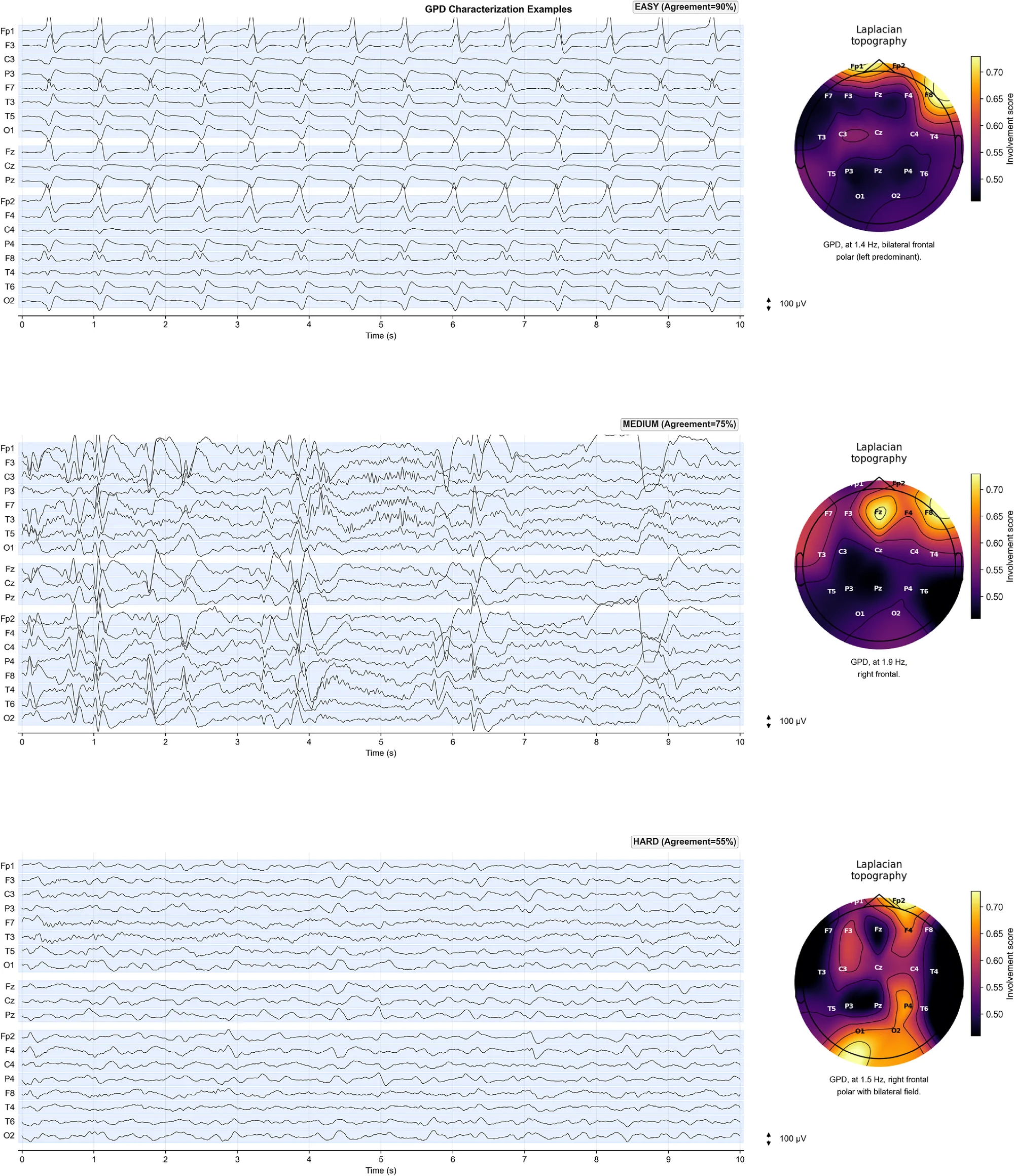
GPD characterization examples at three difficulty levels. Three representative generalized periodic discharge (GPD) cases, following the same format as figure 6. Discharge markers span both hemispheres, reflecting the generalized distribution. Scale bar: 100 μV.

**Figure 8.**
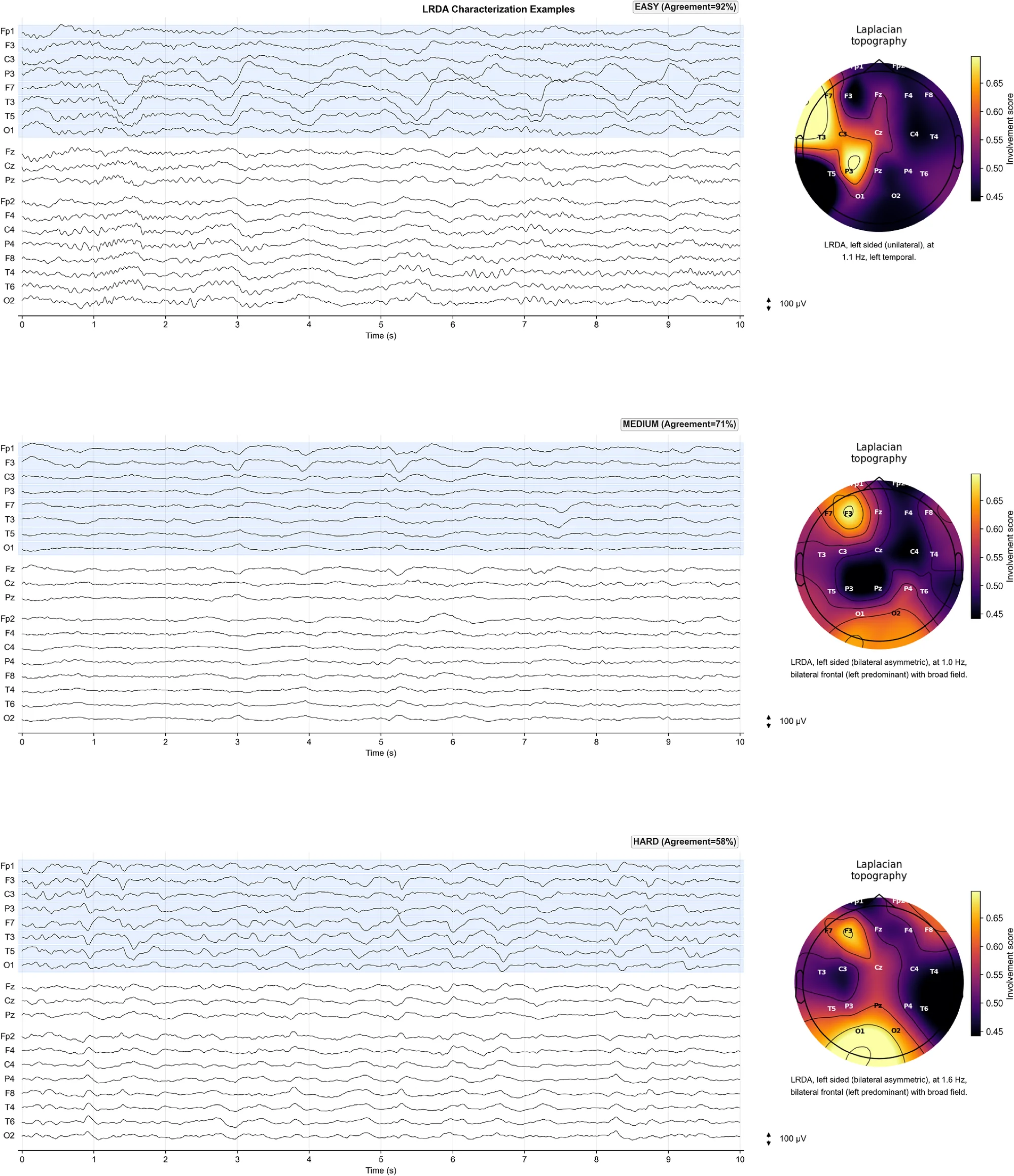
LRDA characterization examples at three difficulty levels. Three representative lateralized rhythmic delta activity (LRDA) cases, following the same format as figure 6. No discharge markers are shown. Light blue shading indicates the dominant hemisphere. Scale bar: 100 μV.

**Figure 9.**
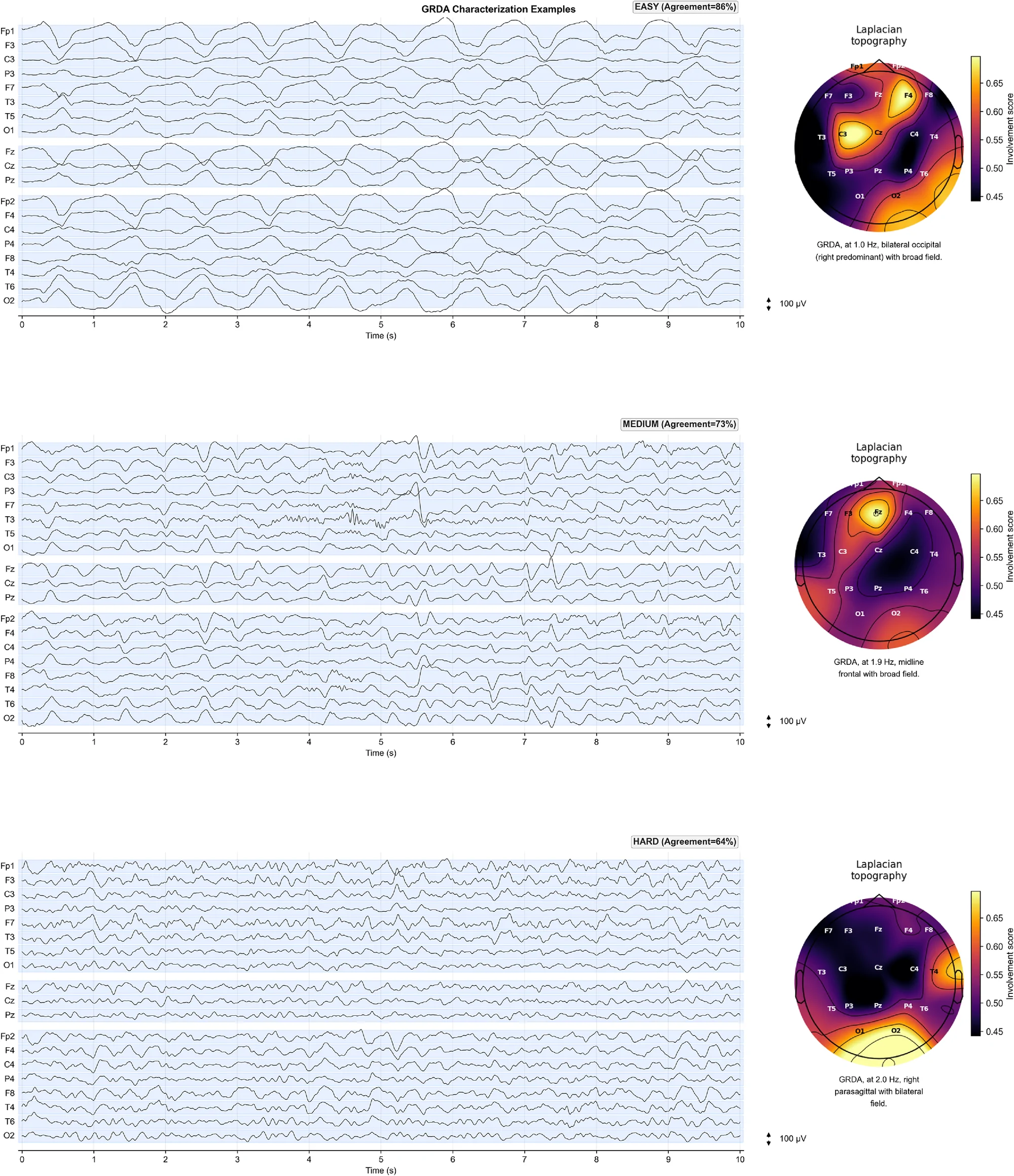
GRDA characterization examples at three difficulty levels. Three representative generalized rhythmic delta activity (GRDA) cases, following the same format as figure 6. No discharge markers are shown. Light blue shading covers all channels, reflecting the generalized distribution. Scale bar: 100 μV.

### 3.6. Model architecture comparison

A comparison of neural architecture strategies (table 10) confirmed the value of combining learned evidence with structured inference. The production HemiCET-UNet + DP pipeline (*F*1 =0.817, frequency ρ=0.837) substantially outperformed an end-to-end CNN approach (E2E-CNN; *F*1 =0.006, frequency ρ=0.001), which failed completely with the available training data size of 657 labeled examples. With current data volumes, end-to-end neural models could not learn the temporal structure that DP encoded as a prior. The winning strategy combined neural evidence generation (HemiCET-UNet learning what a discharge looks like) with structured inference (DP learning when discharges should occur, given periodicity constraints).

**Table 10.** Structured inference versus end-to-end learning on the discharge-timing task, at the current training-set size of 657 labeled segments. Both models were trained and evaluated under the same 5-fold patient-stratified cross-validation and are scored on the same 576 evaluation segments with the same ±100 ms matching tolerance as table 8. The production model generates learned per-frame evidence with HemiCET-UNet and then imposes an approximately-periodic temporal prior by dynamic programming; the end-to-end model regresses discharge times directly from the EEG with no structural prior. The end-to-end model did not learn the task at this data scale (*F*1 =0.006), which is the empirical basis for the hybrid design argued for in section 4.3. Abbreviations: DP, dynamic programming; E2E, end-to-end; *F*1, *F*1 score; ρ, Spearman rank correlation.

Model	*F*1	Freq ρ	Notes
HemiCET-UNet + DP (production)	0.817	0.837	Neural evidence + DP
E2E-CNN (direct prediction)	0.006	0.001	Failed with ∼657 training examples

### 3.7. Montage ablation

Varying only the input montage (section 2.2; full results in supplementary section S3), the production bipolar derivation was the best-performing input on every RDA task tested. This held specifically on GRDA, the generalized and spatially synchronous subtype for which common-mode subtraction would be expected to be most damaging: bipolar reached a mean EA ICC of 0.930 against an EE ceiling of 0.946, while common-average monopolar reached 0.901, the surface Laplacian 0.854 (Δ=−0.076, p<0.001), and the two fusion conditions 0.893 and 0.894 (both p<0.001). The same ordering held on LRDA frequency and on LRDA laterality. Fusing bipolar with monopolar or Laplacian channels did not improve any *RDA* task; the single numerical gain anywhere in the RDA ablation ($\Delta\kappa = +0.015$ for bipolar + monopolar on LRDA laterality) was not significant (p=0.730).

The corresponding ablation for the PD-Profiler tells a more task-dependent story and is reported in full in supplementary section S3. There, on GPD frequency—the generalized subtype, where common-mode subtraction was predicted to matter most—every alternative montage scored above bipolar, with bipolar + monopolar fusion highest (Spearman ρ 0.714 vs. 0.631). That difference did not reach significance on the rank correlation under a patient-stratified bootstrap ($\Delta\rho = +0.083$, 95% CI [−0.001,+0.139], p=0.069), though the same comparison was significant on MAE (Δ=0.029 Hz, p<0.001). The advantage did not transfer: the same alternatives were significantly *worse* on LPD frequency and on PD hemisphere lateralization, the latter being a production output on which bipolar was the best input (AUC 0.988, every alternative lower). We therefore retain the bipolar derivation across both pipelines, while noting that the mechanism the reviewer identified is visible in the PD data and that a task-specific input for GPD frequency is a concrete direction for future work. A plausible reading is that what the RDA estimator must recover is the *frequency* of the rhythm rather than its amplitude, and the bipolar derivation’s rejection of reference activity and low-frequency drift improves the signal-to-noise ratio of the instantaneous-phase estimate by more than common-mode subtraction costs it. We therefore retain the bipolar derivation, now on measured rather than assumed grounds.

## 4. Discussion

### 4.1. Summary of contributions and significance

The vision motivating this work is to make quantitative characterization of periodic and rhythmic EEG patterns—their frequency, spatial localization, lateralization, and individual discharge timing—as accurate and reproducible as the binary detection step that currently dominates automated cEEG analysis. If characterization at expert-level reliability can be automated, then large-scale studies of how the *features* of epileptiform activity, not merely its presence, predict and contribute to neurological injury become feasible—opening the door to more refined dose–response analyses and, ultimately, to characterization-stratified clinical trials of antiseizure treatment. To pursue this vision we framed PD and RDA characterization as four interrelated subtasks (lateralization, spatial localization, frequency, and individual discharge timing), systematically compared more than 300 candidate algorithm variants in a contest-of-methods framework, combined the winning components into two integrated pipelines (PD-Profiler and RDA-Profiler), and benchmarked the result against multi-rater expert annotations using the same statistical framework as the prior state of the art [25]. The resulting system exceeded the four-rater EE ceiling at significance on LPD frequency (mean EA ICC 0.931 vs. EE 0.897, Δ=+0.034, p<0.001), and was statistically indistinguishable from EE on every other attribute tested (GPD frequency p=0.061, GRDA frequency p=0.056, LRDA frequency p=0.435, LPD laterality p=0.079, LRDA laterality p=0.269), with no attribute falling significantly below the ceiling. The V14 amplitude/rhythmicity hybrid laterality rule—which defaults to the V12 amplitude call but overrides it when four amplitude-normalized rhythmicity measures unanimously disagree—was what closed the LRDA-laterality gap: under the amplitude-only rule the algorithm sat significantly below the ceiling (mean EA κ 0.912, Δ=−0.081, p=0.035), and under V14 it does not (mean EA κ 0.961, Δ=−0.032, p=0.269). LRDA laterality and LRDA frequency nonetheless remain the two attributes with the largest residual distance from the expert ceiling, and are the natural next targets. The system achieved frequency-estimation Spearman correlations 1.6 to 9.6 fold higher than the prior signal-processing baseline (paired-bootstrap p<0.001 on every subtype; table 6). To our knowledge, this is the first automated system to reach expert-level IRR on *any* characterization-task attribute for periodic or rhythmic critical-care EEG patterns. The specific contributions are: (i) we *present* a hybrid PD characterization architecture that combines learned neural evidence (HemiCET-UNet) with structured dynamic-programming inference, and *demonstrate* that it outperforms both purely handcrafted and purely end-to-end approaches at current dataset scales; (ii) we *introduce* discharge-locked topographic localization, a principled spatial-localization method that bypasses noisy expert spatial-extent labels by computing the mean voltage topography at the moment of each detected discharge; (iii) we *worked out* a contest-of-methods development framework in which more than 300 candidate algorithms were systematically implemented and benchmarked, providing a reproducible record of which design choices mattered; and (iv) we *benchmark* all characterization tasks against multi-rater expert annotations and IRR ceilings, enabling direct comparison of algorithm–expert agreement with EE agreement. The remainder of this section discusses comparison with prior work (section 4.2), the lessons we draw from learned versus handcrafted features and from label-quality discoveries (sections 4.3 and 4.4), the clinical implications (section 4.5), and the system’s limitations and future work (section 4.6).

### 4.2. Comparison with prior work

The most directly comparable prior work is that of Tăuţan *et al* [25], who described a signal-processing approach to PD and RDA characterization using autocorrelation-based frequency estimation and spectral spatial analysis. The present system achieved substantially higher performance across all characterization tasks (table 6). For frequency estimation on the canonical labeled cohort, the improvement in Spearman correlation ranged from 1.6-fold on GPD (ρ 0.802 vs. 0.508) to 9.6-fold on LRDA (ρ 0.740 vs. 0.077), with intermediate gains on LPD (2.8-fold) and GRDA (6.0-fold), and approximately a two- to three-fold reduction in MAE across subtypes; all four $\Delta\rho$ comparisons reached p<0.001 under the paired patient-stratified bootstrap (table 6). For spatial extent, the present system achieved ICC values comparable to but not exceeding EE agreement (PD: 0.764 vs. expert 0.845, tied with Tautan; RDA: 0.371 vs. expert 0.373), while Tautan *et al* achieved lower ICC values for RDA (0.215). On PD specifically, the Jaccard analysis at the optimized binarization threshold (table 9) is a more reliable spatial summary—the PD-Profiler reached 97.3% of EE Jaccard agreement; the lower ICC reflects pooling LPD and GPD, the latter of which has very weak inter-expert spatial agreement on its own. As discussed below (section 4.4), we therefore treat the discharge-locked topographic map as the principal spatial output of the system rather than the categorical channel-fraction.

Several factors contributed to these improvements. First, the use of learned evidence (HemiCET-UNet) rather than purely handcrafted features enabled the PD-Profiler to capture discharge morphology patterns that are difficult to specify algorithmically. Second, the IPI-derived frequency from detected discharge timing provided a more direct and accurate frequency estimate than spectral methods, which are sensitive to harmonics, non-stationarity, and contamination from non-periodic activity. Third, the iterative narrowband refinement in the RDA-Profiler (NB-Hilbert) leveraged the estimated frequency to isolate the pattern of interest before computing lateralization and refined frequency estimates, reducing the influence of confounding signals. Fourth, the PLV approach to spatial extent quantified the functional coherence of each channel with the dominant pattern, providing a more principled metric than amplitude-based channel counting.

No prior work has reported automated discharge-level timing for individual PDs or BIPD detection, making direct comparison on these tasks impossible. McGraw *et al* [16] described an automated method for quantifying *burst-level* PD activity, but did not address per-discharge timing, lateralization, or BIPD detection. The present *F*1 of 0.817 for discharge detection establishes a baseline benchmark for future studies; the BIPD-vs.-GPD AUC of 0.937 is reported here as a preliminary signal—the held-out BIPD evaluation set contains only 10 confirmed cases and a definitive comparison will require multi-site case collection (section 4.6).

### 4.3. Learned evidence versus handcrafted features

A key finding of this work: when both methods used the same DP inference framework, the learned HemiCET-UNet evidence trace (a 1D U-Net operating on single channels) outperformed handcrafted features (pointiness + TKEO) substantially. HemiCET-UNet + DP reached *F*1 =0.817 versus the per-hemisphere handcrafted baseline’s *F*1 =0.586—a 39% improvement. More strikingly, learned evidence with imperfect CNN+ACF frequency exceeded the Oracle configuration that used expert-annotated frequency with handcrafted evidence (*F*1 =0.817 vs. 0.578), showing that the quality of discharge evidence mattered more than the accuracy of the frequency prior.

This result supports a hybrid architecture in which neural networks handle perceptual tasks (recognizing discharge morphology in raw EEG) while domain knowledge encodes structural constraints (periodicity in the DP algorithm). Pure end-to-end approaches, which must learn both the perceptual and structural components from labeled data, failed at the current training set size of 657 labeled segments. This finding is consistent with the broader machine learning literature on combining neural perception with model-based reasoning for structured prediction tasks [26], and parallels the long-standing success of hybrid neural-plus-structured-decoding architectures in adjacent biomedical signal-processing problems where labeled data are similarly limited and the underlying signal has strong temporal structure—most notably automatic speech recognition, which routinely combines a learned acoustic model with a structured decoder.

The complementary value of handcrafted and learned evidence was confirmed by the product-boost combination formula, which yielded better performance than either source alone. The handcrafted pointiness trace captured sharp transients that the HemiCET-UNet occasionally missed (particularly low-amplitude discharges in noisy channels), while the HemiCET-UNet identified discharges with atypical morphology that did not trigger the pointiness detector.

### 4.4. Label quality discovery

Apparent algorithm limitations repeatedly traced to label noise rather than algorithm deficiency. Three examples illustrate this phenomenon.

First, the original per-patient labels aggregated IIIC crowd votes across all 10 s segments within a 10-minute recording, such that a patient labeled ‘LRDA’ might have had most individual segments voted as ‘other’ by the crowd. Migration to per-segment labels improved RDA lateralization AUC from 0.58 to 0.84 using the same algorithms.

Second, MW’s systematic frequency review of 993 RDA segments identified that the NB-Hilbert algorithm estimate matched the expert exactly in 59% of cases and was within 0.25 Hz in 85% of cases. Among the 399 corrections, many involved ambiguous segments where frequency estimation was inherently uncertain.

Third, in a post-hoc analysis of PD frequency discrepancies exceeding 0.5 Hz, MW re-reviewed all discordant cases and judged the algorithm estimate to be correct (more consistent with the visual pattern) in 94% of cases, leading to ground-truth label corrections. These findings underscore the importance of iterative, bidirectional quality improvement between algorithm development and annotation refinement.

The same logic applies to the RDA spatial-extent task, where the absolute EE ICC of ≈0.37 is striking: when three trained electroencephalographers reach only that level of agreement on a labeling task, the task itself—region-counting from a verbal/categorical description of field distribution—is intrinsically noisy, and chasing a 0.37-ceiling target with an ever-more-elaborate algorithm yields rapidly diminishing returns. Our response was to keep channel-wise spatial-extent fractions in the system as a backwards-compatibility output (so that an ACNS 2021 verbal description can still be produced) but to designate the discharge-locked topographic map (section 2.4) as the principal spatial output of the system. The topographic map is, by construction, free of the verbal-description ambiguity that produces the low spatial-extent ICC: it is a reproducible voltage image of where the cortical generators of the discharge actually are, computed deterministically from the detected discharge times and the same EEG. We therefore consider quantitative spatial information about PDs and RDA to be *better* captured by the topographic output than by the spatial-extent fraction, and recommend that downstream users (clinical and research) adopt the topographic map as the primary spatial readout.

### 4.5. Clinical implications

The intended clinical contribution of this system is *speed via automation*, not better individual clinical decisions. Current practice—visual review of long cEEG recordings followed by dictated reports describing periodic and rhythmic patterns—is bottlenecked by two problems we directly target: (i) low between-expert reliability on several characterization attributes (frequency, laterality, and spatial extent), which we mitigate by replacing subjective judgments with reproducible algorithmic outputs that match or exceed the EE ceiling on the canonical 4-rater cohort (figure 4); and (ii) the slowness of de novo expert characterization, which we mitigate by producing a complete ACNS 2021-aligned structured description (lateralization, spatial localization, frequency, per-discharge timing for PDs, and a draft BIPD label where applicable) that the reviewing electroencephalographer can sign off on or revise rather than write from scratch. We do not claim that automated characterization makes individual decisions *clinically safer* than expert visual review by an experienced electroencephalographer; the appropriate framing is that the system replaces the time-consuming, low-reliability *characterization* step in the existing workflow without altering the higher-level diagnostic and treatment-management decisions that follow. All algorithmic outputs are intended as a starting point for expert sign-off, not as autonomous clinical decisions.

A second, downstream benefit is that standardized quantitative characterization at scale enables population-level epidemiologic and outcome studies of how the *features* of periodic and rhythmic activity (rather than just their presence) predict and contribute to neurological injury—studies that are currently bottlenecked by the labor intensity of manual annotation. Third, the discharge-locked topographic maps (section 4.4) provide a reproducible voltage image of cortical generator location that is more objective than verbal descriptions of field distribution and that downstream analyses can use directly.

**Failure modes and where expert review remains essential.** The independent-expert evaluation (section 3.2, figure 4) shows where the algorithm tracks experts well and where it does not. The system reaches or exceeds the EE ceiling on PD frequency and is statistically indistinguishable from it on RDA frequency and on LPD/LRDA laterality, but a small absolute MAE bias on RDA frequency (∼0.05–0.06 Hz) remains; for clinical use, this implies that frequency reads near canonical pattern boundaries (e.g. ∼2.5 Hz on the seizure/non-seizure border) should still be confirmed visually. The LPD-vs.-LRDA boundary (section 3.1) and the BIPD-vs.-GPD boundary (section 2.6, table 5) are the two most consequential boundary cases: the former because LPD and LRDA can have very similar amplitude/rhythmicity profiles in slowly evolving patterns, and the latter because BIPD has different prognostic implications than synchronous GPD and our held-out BIPD evaluation set (n=10) is too small for the AUC point estimate to be definitive. Very low-amplitude patterns and very low-rate (<0.5 Hz) patterns are also at the edge of where the present system has been characterized and should be re-reviewed by an electroencephalographer when the per-channel CNN probabilities or the narrowband peak strength flag the segment as low-confidence. We therefore recommend an *expert-in-the-loop* deployment: the system produces a complete ACNS 2021 description as a draft, and the reviewing electroencephalographer accepts, edits, or rejects each field before sign-off.

The discharge timing capability (*F*1 =0.817, timing MAE =3.1 ms) enables downstream analyses that were previously impractical at scale, including IPI variability assessment (relevant to the ACNS ‘nearly regular’ criterion), discharge-locked averaging for morphology analysis, and temporal relationship analysis between discharge patterns and other physiological signals.

**Deployment architecture: pattern classification upstream, characterization downstream.** The pipeline as evaluated above takes pre-classified 10 s segments (LPD/GPD/LRDA/GRDA) as input, which understates the deployment story and is worth making explicit. The intended production architecture pipes *Morgoth* [22]—our existing pattern-classification system, trained on the IIIC consensus labels and shown there to exceed expert agreement at separating LPD, GPD, LRDA, GRDA, seizure, and ‘other’ from each other—into the present characterization system. Morgoth segments a continuous cEEG record into 10 s epochs and assigns each one of the six IIIC class labels; the LPD and GPD epochs are then routed through the PD-Profiler (section 2.4), the LRDA and GRDA epochs through the RDA-Profiler (section 2.5), and within the PD-Profiler the BIPD module (section 2.6) flags borderline segments whose bilateral discharge timing is consistent with independent rather than synchronous PDs. Morgoth currently labels epileptiform-appearing independent PDs as GPDs by default, so re-classification of borderline GPD epochs as BIPD is one of the small set of class-boundary corrections the new system can contribute back to the upstream classifier. End-to-end characterization performance under this pipeline (Morgoth → Profiler, with classification errors propagating into characterization errors) is left for a follow-up evaluation, since the present paper is scoped to the characterization step. The point of comparison for that future evaluation, however, is not a hypothetical perfect classifier but current clinical practice—visual review of the same segments—which is itself bottlenecked by inter-rater disagreement at the same class boundaries (LPD-vs.-LRDA, BIPD-vs.-GPD, low-amplitude-vs.-other) that the integrated system has to navigate.

### 4.6. Limitations and future work

First, the BIPD evaluation was limited by the rarity of the pattern. Only 21 confirmed BIPD segments were identified across an institutional cEEG database covering thousands of patients (10.6% yield from the 198 candidate segments screened), with 11 used for training and 10 retained as a held-out evaluation set. CIs on the BIPD AUC point estimates are accordingly wide (table 5), and the headline 0.937 BIPD-vs.-GPD AUC and 0.862 3-way macro AUC should be read as preliminary signals that the synthetic-data training procedure produces a non-trivial classifier, not as a definitive characterization of in-the-wild BIPD detection performance. The synthetic-to-real transfer assumption—that pairs of independent LPD sequences from different patients adequately reproduce the timing structure of real BIPD—is plausible given the timing features the classifier relies on, but warrants direct verification on a substantially larger real-BIPD cohort. Such a cohort is not realistically obtainable from a single institution given the rarity of the pattern; we are therefore pursuing multi-site collection through a planned 50+ hospital consortium, with a separate BIPD-focused evaluation as the intended follow-up. The current BIPD module should not be deployed as an autonomous clinical decision aid until that evaluation has been completed.

Second, the per-segment outputs of the system as described here are point estimates without calibrated uncertainty: the algorithm reports a single frequency in Hz, a single laterality call (left/right/bilateral), a single discharge sequence, and a fraction of involved channels, with no ‘low-confidence’ marker that distinguishes a prototypical segment from an ambiguous one. The headline cohort-level performance metrics are reported with 95% CIs (tables 5–7, figure 4), but individual segments do not yet carry analogous uncertainty estimates. The system already produces the raw quantities needed to construct calibrated per-segment uncertainty—the standard deviation of the inter-discharge intervals (which determines the SE of the IPI-derived frequency), the per-channel ChannelPD-Net probability dispersion across hemispheres (which determines the confidence of the laterality call), the narrowband peak prominence and *Q*-factor (which flag low-rhythmicity segments), and the cross-fold variance of the 5-fold CNN ensemble. Combining these into a single calibrated, end-user-facing confidence band on each output (and an explicit ambiguity flag when multiple signals jointly indicate low confidence) is left to follow-up work; in the interim, we recommend treating any segment with diffuse per-channel CNN probabilities, a low narrowband peak prominence, or unusually high IPI variance as low-confidence and re-reviewing it visually.

Third, RDA wave timing (identifying the onset, peak, and offset of each rhythmic delta wave cycle) was not addressed in this work. While 502 segments have been labeled with wave timing annotations, the automated method for wave detection remains under development. Wave-level timing would enable phase-locked analyses (e.g. whether fast activity occurs at a specific RDA phase) and wave-by-wave frequency estimation.

Fourth, the discharge-locked topographic localization approach, while providing a principled and ground-truth-free spatial representation, has not been formally validated against a gold standard. The Jaccard analysis of spatial extent (table 9) demonstrated near-expert agreement for threshold-based channel involvement, but the topographic maps themselves were evaluated only qualitatively through expert visual review. Future work should develop quantitative validation methods, potentially using intracranial EEG recordings as ground truth.

Fifth, the development and evaluation cohorts were drawn from a single institution. Although the cohort spans thousands of patients across multiple ICUs and a wide range of clinical contexts, both training and evaluation use the same site’s expert rater pool. Patient-stratified cross-validation rules out leakage of individual patients between training and evaluation but does not, by itself, address site-level generalization. The factors that most plausibly differ across sites are upstream of the per-segment input we operate on (variation in patient mix, indication for cEEG monitoring, and labeling conventions among reading electroencephalographers), rather than in the EEG signal itself: electrode placement (10–20 system), reference montage, and acquisition setup are standardized across ACNS-aligned acute-care laboratories, and the input pipeline ingests standard 10 s 19-channel average-reference segments at 200 Hz with no site-specific assumptions. We therefore expect the algorithm’s behavior to be qualitatively similar on data from other acute-care EEG services, but a formal multi-site validation remains a necessary step before clinical deployment and is part of the same consortium effort described above for BIPD case collection.

Sixth, the system operates on isolated 10 s EEG segments and does not model temporal evolution of patterns across longer recordings. Clinical monitoring generates hours to days of continuous data, and characterization of pattern evolution (e.g. frequency trending, spatial spread) would enhance clinical utility. Integration with existing pattern detection systems (e.g. Morgoth [22]) that provide real-time classification of continuous EEG is a natural extension.

Seventh, the current system does not address seizure patterns, which share features with PDs and RDA but require distinct characterization. Extension to the full ACNS critical care EEG terminology, including seizures, brief potentially ictal rhythmic discharges (BIRDs), and stimulus-induced patterns, is an important direction.

Finally, training-data volume remains a constraint for the HemiCET-UNet discharge detector (657 labeled segments). Self-supervised pretraining on unlabeled EEG data and transfer learning from larger datasets may help overcome this limitation.

## 5. Conclusion

This work presents a comprehensive automated system for characterizing PDs and RDA in continuous EEG, addressing lateralization, spatial localization, discharge timing, frequency estimation, and BIPD detection within a unified framework. The PD-Profiler combines learned neural evidence (HemiCET-UNet) with structured dynamic programming inference, while the RDA-Profiler uses iterative narrowband signal processing with phase-coherence analysis. Across all characterization tasks, the system achieves performance approaching or matching expert inter-rater agreement, with frequency estimation correlations 1.6- to 9.6-fold higher than the previous state of the art (table 6).

The key technical insight is this: hybrid architectures outperform both purely handcrafted and purely end-to-end approaches at current data scales. Such architectures combine neural networks for perceptual feature extraction with domain-knowledge-encoded temporal priors. The discharge-locked topographic localization method provides a principled, threshold-free spatial representation that directly computes the voltage field distribution at discharge peaks. The iterative, bidirectional relationship between algorithm development and annotation refinement—where algorithm predictions reveal label errors and corrected labels improve algorithm evaluation—was essential to achieving reliable performance benchmarks.

The system produces ACNS 2021-formatted verbal descriptions suitable for clinical reporting and provides a foundation for large-scale quantitative analysis of periodic and rhythmic EEG patterns. We have made all code and evaluation scripts publicly available.

## Data Availability

The data that support the findings of this study are openly available at the following URL/DOI: https://github.com/bdsp-core/grond [27]. Algorithm methods and contests available at: https://doi.org/10.1088/1741-2552/aeb331/data1. **Code.** The source code for the GROND system—both pipelines, all algorithm contest entries, the montage-ablation scripts, and the figure- and table-generation scripts used to produce this manuscript—is openly available at: https://github.com/bdsp-core/grond. The repository includes REPRODUCIBILITY.md, which maps every figure and table to the script that regenerates it, and paper_materials/generate_all_figures.py, which regenerates the complete figure set from a single entry point. Trained model weights for ChannelPD-Net, HemiCET-UNet and the BIPD classifier are distributed with the data (below). **Data.** The EEG segments, expert-rater label files, algorithm predictions, cohort flags and trained model weights are released through the Brain Data Science Platform (https://bdsp.io) as the project ‘GROND: Automated Characterization of Periodic and Rhythmic EEG Patterns’. The data are hosted in the credentialed BDSP Amazon S3 bucket under the prefix s3://bdsp-opendata-credentialed/grond/ (2.0 GB) and comprise: a single self-contained HDF5 bank, grond_data.h5, holding all 14 307 ten-second EEG segments as 19-channel monopolar referential recordings at 200 Hz together with every label, algorithm prediction and cohort flag keyed by segment identifier; the trained model weights under data/; and the four self-contained HTML review tools under independent_expert_tasks/ that the independent expert raters used, so that the exact rating interface behind the IRR results can be inspected and re-run. After credentialing, the data can be retrieved with aws s3 sync s3://bdsp-opendata-credentialed/grond/ ./grond-data/ **Access conditions.** The EEG data are de-identified (surrogate patient identifiers and shifted dates) but remain governed by the BDSP Data Use Agreement, so access is credentialed rather than open: investigators register at https://bdsp.io, complete human-subjects research training, and sign the Data Use Agreement. The code carries no such restriction. Questions about data access may be directed to support@bdsp.io.
